# Electrocatalytic C(sp^3^)–H/C(sp)–H cross-coupling in continuous flow through TEMPO/copper relay catalysis

**DOI:** 10.3762/bjoc.17.178

**Published:** 2021-10-28

**Authors:** Bin Guo, Hai-Chao Xu

**Affiliations:** 1 Key Laboratory of Chemical Biology of Fujian Province and College of Chemistry and Chemical Engineering, Xiamen University, People’s Republic of China

**Keywords:** continuous flow, copper, catalysis, dehydrogenative cross-coupling, electrochemistry

## Abstract

Electrocatalytic dehydrogenative C(sp^3^)–H/C(sp)–H cross-coupling of tetrahydroisoquinolines with terminal alkynes has been achieved in a continuous-flow microreactor through 2,2,6,6-tetramethylpiperidine 1-oxyl (TEMPO)/copper relay catalysis. The reaction is easily scalable and requires low concentration of supporting electrolyte and no external chemical oxidants or ligands, providing straightforward and sustainable access to 2-functionalized tetrahydroisoquinolines.

## Introduction

**Scheme 1:**
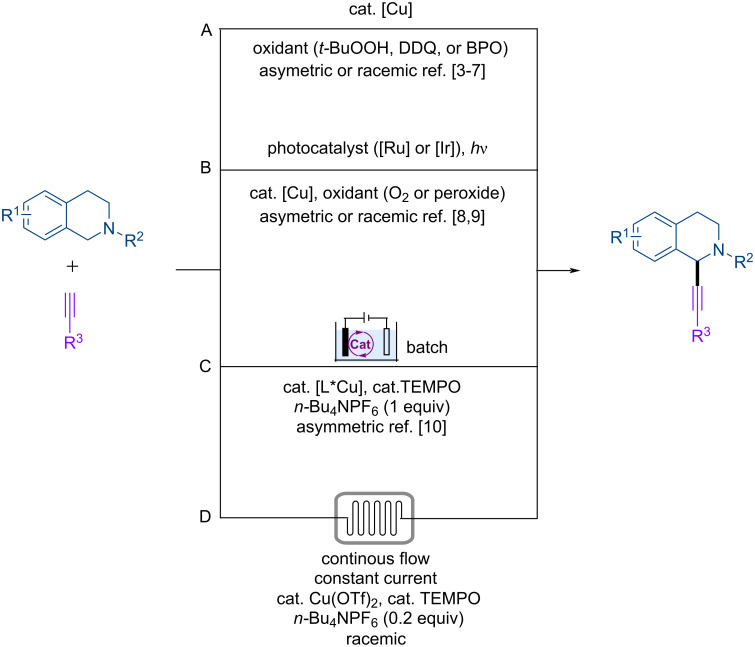
C(sp^3^)–H alkynylation of tetrahydroisoquinolines. L* = chiral ligand. TEMPO = 2,2,6,6-tetramethylpiperidine 1-oxyl. DDQ = 2,3-dichloro-5,6-dicyano-1,4-benzoquinone. BPO = benzoyl peroxide.

The dehydrogenative cross-coupling of two C–H bonds represents an ideal strategy for the construction of C–C bonds [1–2]. In this context, few methods have been developed for the dehydrogenative cross-coupling of tetrahydroisoquinolines with terminal alkynes because of the prevalence of the tetrahydroisoquinoline moiety in natural products and bioactive molecules [3–10]. These methods proceed through the oxidation of the tetrahydroisoquinoline to an iminium intermediate with various chemical oxidants such as peroxides and DDQ followed by reaction with the copper acetylide species to deliver the 2-substituted tetrahydroisoquinoline product (Scheme 1A). These methods usually require elevated temperatures [3–5], prompting the development of mild conditions by merging photoredox catalysis with copper catalysis (Scheme 1B) [8–9]. Notwithstanding of these outstanding achievements, noble metal-based catalysts and chemical oxidants are employed under these photochemical conditions.

Organic electrochemistry is an ideal tool for promoting dehydrogenative cross-coupling reactions as no external chemical oxidants are needed [11–19]. In this context, Mei and co-workers have reported an elegant TEMPO/[L*Cu] co-catalyzed asymmetric electrochemical dehydrogenative cross-coupling reaction of tetrahydroisoquinolines with terminal alkynes (Scheme 1C) [10]. The chiral ligand was found to be critical for the stereoinduction as well as product formation for these electrochemical reactions that are conducted in batch. Continuous-flow electrochemical microreactors offer several advantages for electrosynthesis and have been employed to reduce the use of supporting electrolyte, facilitate reaction scale-up, and increase reaction efficiency [20–32]. Despite these advantages of continuous-flow electrosynthesis and the intense interests in transition-metal electrocatalysis [33–39], transition-metal electrocatalysis in continuous flow remains underexplored [40]. With our continued interests in transition-metal electrocatalysis [41–42] and continuous-flow electrosynthesis [43–48], we report herein the electrocatalytic dehydrogenative cross-coupling reaction of tetrahydroisoquinolines with terminal alkynes in continuous flow (Scheme 1D). These reactions require low loadings of supporting electrolyte and proceed through Cu/TEMPO relay catalysis without need for additional ligands.

## Results and Discussion

The electrosynthesis was conducted in a microreactor equipped with two Pt electrodes as the anode and cathode and operated with a constant current (Table 1). Under the optimized conditions, a solution of tetrahydroisoquinoline **1a** (1 equiv), alkyne **2** (1.5 equiv), Cu(OTf)_2_ (10 mol %), TEMPO (20 mol %), *n-*Bu_4_NPF_6_ (0.2 equiv), and TFE (3.5 equiv) in MeCN was passed through the cell at 0.2 mL min^−1^ to give the desired product **3** in 86% yield (Table 1, entry 1). Pleasingly, a good yield of 82% was obtained in the absence of supporting electrolyte (Table 1, entry 2). While product formation was observed without TEMPO (Table 1, entry 3) and TFE (Table 1, entry 4), albeit in low yields, the reaction failed completely without the copper salt (Table 1, entry 5). Other variations also resulted in diminished yield of **3**, such as lowering the loading of Cu(OTf)_2_ to 5 mol % (Table 1, entry 6), replacing Cu(OTf)_2_ with other copper salts such as Cu(acac)_2_ (Table 1, entry 7), Cu(TFA)_2_, (Table 1, entry 8), Cu(OAc)_2_ (Table 1, entry 9) and replacing TFE with other protic additives including MeOH (Table 1, entry 10), EtOH (Table 1, entry 11), HFIP (Table 1, entry 12) and H_2_O (Table 1, entry 13).

**Table 1:** Optimization of reaction conditions.^a^

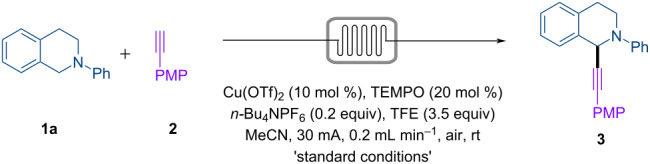		

Entry	Deviation from standard conditions	Yield of **3** (%)

1	none	86^b^
2	no *n-*Bu_4_NPF_6_	82
3	no TEMPO	35
4	no TFE	19
5	no Cu(OTf)_2_	0
6	Cu(OTf)_2_ (5 mol %)	71
7	Cu(acac)_2_ instead of Cu(OTf)_2_	17
8	Cu(TFA)_2_ instead of Cu(OTf)_2_	77
9	Cu(OAc)_2_ instead of Cu(OTf)_2_	40
10	MeOH instead of TFE	60
11	EtOH instead of TFE	50
12	HFIP instead of TFE	38
13	H_2_O instead of TFE	20

^a^Standard conditions: **1a** (0.21 mmol), **2** (0.32 mmol, 1.5 equiv), MeCN (7 mL), Pt anode, Pt cathode, interelectrode distance = 0.25 mm, 3.1 F mol^−1^. Yield of product **3** is determined by ^1^H NMR analysis using 1,3,5-trimethoxybenzene as the internal standard. TFE, 2,2,2-trifluoroethanol. PMP, *p*-methoxyphenyl. HFIP, 1,1,1,3,3,3-hexafluoropropan-2-ol. TEMPO, 2,2,6,6-tetramethylpiperidine 1-oxyl. Cu(acac)_2_, Copper(II) acetylacetonate. Cu(TFA)_2_, Copper(II) trifluoroacetate. ^b^Isolated yield.

**Scheme 2:**
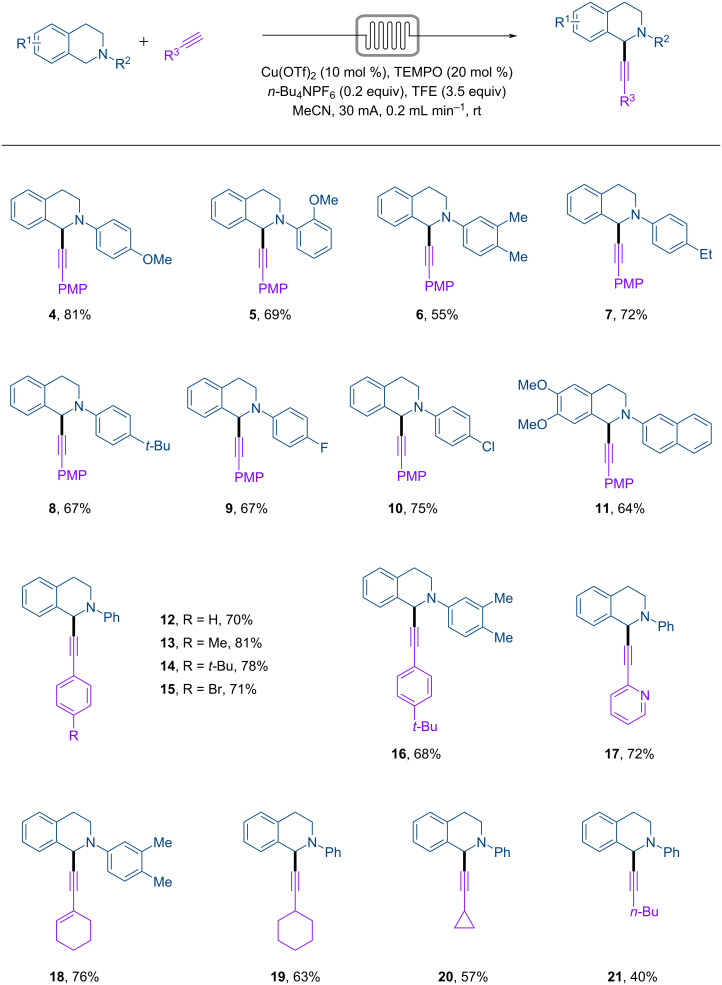
Substrate scope. Reaction conditions: Pt anode, Pt cathode, interelectrode distance 0.25 mm, **1** (0.03 M, 0.21 mmol), **2** (0.045 M, 1.5 equiv), Cu(OTf)_2_ (10 mol %), TEMPO (20 mol %), *n-*Bu_4_NPF_6_ (20 mol %), TFE (3.5 equiv), MeCN (7 mL), I = 30 mA, flow rate = 0.20 mL min^−1^, rt. Isolated yields are reported.

The scope of the continuous-flow electrosynthesis was investigated by varying the substituents of the tetrahydroisoquinoline and the alkyne (Scheme 2). The *N*-phenyl ring of the tetrahydroisoquinoline could be substituted with groups such as OMe (**4**, **5**), Me (**6**), Et (**7**), *t-*Bu (**8**), F (**9**), and Cl (**10**). An *N*-2-naphthalenyl-substituted tetrahydroisoquinoline bearing two OMe groups at 6,7-positions (**11**) also reacted successfully. The alkyne coupling partner also tolerated variation. The reactions were found to be compatible with arylalkynes such as phenylacetylenes bearing at the *para* position a H (**12**), Me (**13**), *t-*Bu (**14**, **16**), or Br (**15**), 2-ethynylpyridine (**17**), alkenylalkynes (**18**), and alkylalkynes (**19**–**21**).

**Scheme 3:**
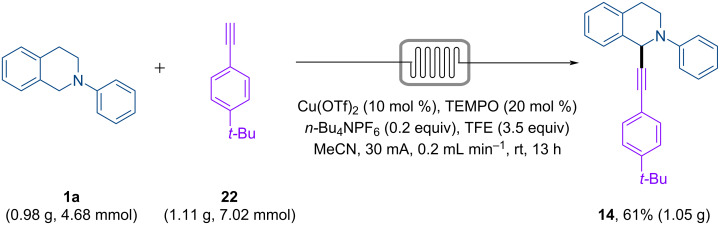
Reaction scale-up.

The continuous-flow electrosynthesis is easily scaled up by passing more material through the reactor [43,49]. Hence, repeating the reaction under flow conditions, with a solution containing 0.98 g of tetrahydroisoquinoline **1a** and 1.11 g of alkyne **22** afforded 1.05 g (61%) of product **14** in 13 h (Scheme 3). The productivity could be increased if multiple reactors were employed in parallel [43].

**Scheme 4:**
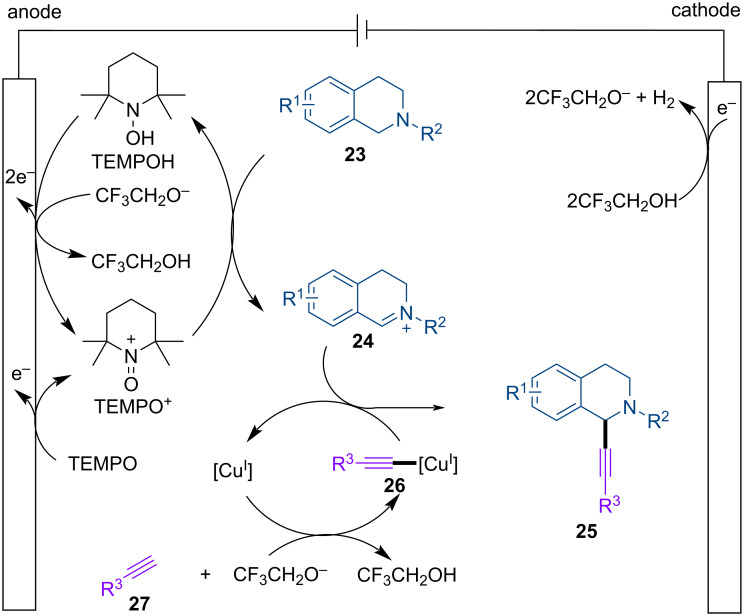
Proposed mechanism.

A mechanism for the electrochemical synthesis was proposed based on reported studies (Scheme 4) [3,10]. Anodic oxidation of TEMPO generates the oxoammonium salt TEMPO^+^ [50–51], which reacts with tetrahydroisoquinoline **23** to generate TEMPOH and iminium ion **24** [52], TEMPOH is oxidized back to TEMPO^+^ on the anode. On the other hand, **24** is converted to the final product **25** through reaction with copper acetylide **26**, which is generated from Cu^I^ and the alkyne **27** with the assistance of CF_3_CH_2_O^−^. The added Cu^II^ precatalyst is likely reduced at the cathode to produce the requisite Cu^I^. The base CF_3_CH_2_O^−^ is produced through cathodic reduction of TFE. The addition of TFE to the reactions helps cathodic H_2_ evolution and may also stabilize the iminium ion through reversible reaction with this cationic species.

## Conclusion

In summary, we have achieved the electrochemical dehydrogenation cross-coupling of tetrahydroisoquinolines with terminal alkynes in continuous flow through Cu/TEMPO relay catalysis. This work demonstrates that continuous-flow electrochemical microreactors can be a viable tool for developing efficient transition-metal electrocatalysis.

## Supporting Information

File 1 General procedure, characterization data for electrolysis products and NMR spectra.
