# Supplementary material for: Electrocatalytic C(sp3)–H/C(sp)–H cross-coupling in continuous flow through TEMPO/copper relay catalysis
Source: Beilstein J Org Chem. 2021 Oct 28;17:2650–6. doi: 10.3762/bjoc.17.178 (PMC8561139; doi:10.3762/bjoc.17.178)
Supplement: File 1 — General procedure, characterization data for electrolysis products and NMR spectra. [file Beilstein_J_Org_Chem-17-2650-s001.pdf]

BEILSTEIN JOURNAL OF ORGANIC CHEMISTRY

Supporting Information

for

Electrocatalytic C(sp^3)–H/C(sp)–H cross-coupling in continuous flow through TEMPO/copper relay catalysis

Bin Guo and Hai-Chao Xu

Beilstein J. Org. Chem. **2021,** *17,* 2650–2656. doi:10.3762/bjoc.17.178

General procedure, characterization data for electrolysis products and NMR spectra

License and Terms: This is a supporting information file under the terms of the Creative Commons Attribution License (https://creativecommons.org/licenses/by/4.0). Please note that the reuse, redistribution and reproduction in particular requires that the author(s) and source are credited and that individual graphics may be subject to special legal provisions.
The license is subject to the *Beilstein Journal of Organic Chemistry* terms and conditions: (https://www.beilstein-journals.org/bjoc/terms)

Table of contents

1. General information S1

2. General procedure for the flow electrolysis S1

3. Procedure for scale up S1

4. Characterization data for the electrolysis products...S2

5. Synthesis and characterization for the 2-aryl-1,2,3,4-tetrahydroiso-quinolines S9

6. Reference S13

7. NMR spectra...S14

1. General information

1,1,1,3,3,3-Hexafluoro-2-propanol (HFIP) and 2,2,2-trifluoroethanol (TFE) were purchased from aladdin. Anhydrous acetonitrile was obtained by distillation from CaH_2/benzophenone. Flash column chromatography was performed with silica gel (100–200, 200–300 mesh). NMR spectra were recorded on Bruker AV-400 and Bruker AV-500 instruments. Data were reported as chemical shifts in ppm relative to $CDCl_3$ (7.26 ppm) for ^1H and $CDCl_3$ (77.2 ppm) for ^{13}C. The abbreviations used for explaining the multiplicities were as follows: s = singlet, d = doublet, t = triplet, q = quartet, m = multiplet, br = broad.

2. General procedure for the flow electrolysis

The electrolysis was conducted using a flow electrolytic cell equipped with a Pt anode and a Pt cathode with a surface area of 10 cm^2 and an interelectrode distance of 250 μm. The solution of tetrahydroisoquinoline (0.03 M, 1.0 equiv), alkyne (0.45 M, 1.5 equiv), $Cu(OTf)_2$ (0.003 M, 10 mol %), TEMPO (0.006 M, 20 mol %), n-Bu_4NPF_6 (0.006 M, 0.2 equiv), and TFE (0.105 M, 3.5 equiv) in dry MeCN was stirred for 120 min at room temperature under air. The reaction solution was pumped *via* a micro syringe pump at a flow rate of 0.2 mL min^{-1} through the flow electrolytic cell operated at a constant current of 30 mA. Once the electrolysis was stable, the outlet solution was collected for 35 min (7 mL). The solution was concentrated under reduced pressure on a rotary evaporator. The residue was chromatographed through silica gel eluting with ethyl acetate/hexanes to give the desired product.

3. Procedure for scale up

The solution of substrate 2-phenyl-1,2,3,4-tetrahydroisoquinoline (**1a**, 0.98 g, 4.68 mmol), 1-(*tert*-butyl)-4-ethynylbenzene (**22**, 1.11 g, 7.02 mmol), $Cu(OTf)_2$ (169.4 mg, 0.47 mmol), TEMPO (146.1 mg, 0.94 mmol), n-Bu_4NPF_6 (362.2 mg, 0.94 mmol), and TFE (1.2 mL) in dry

MeCN (156 mL) was stirred for 240 min at room temperature under air. The reaction solution was pumped *via* a micro syringe pump at a flow rate of 0.2 mL min^{-1} through the flow electrolytic cell operated at a constant current of 30 mA. The combined organic solution was concentrated under reduced pressure on a rotary evaporator. The residue was chromatographed through silica gel eluting with ethyl acetate/hexanes to give **14** in 61% yield (1.05 g).

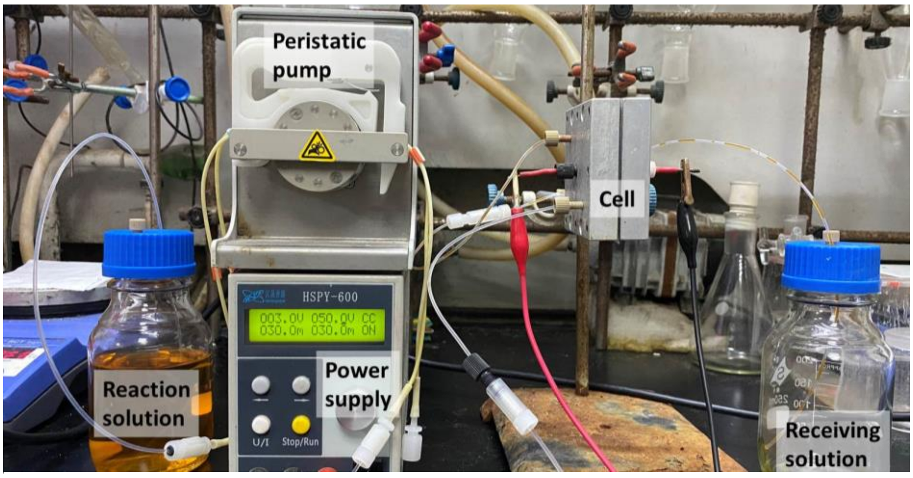

Figure S1. Reaction setup for scale up.

4. Characterization data for the electrolysis products

Note: all the products are known in the literature.

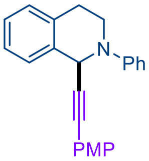

1-((4-Methoxyphenyl)ethynyl)-2-phenyl-1,2,3,4-tetrahydroisoquinoline[1] (**3**). Colorless oil, 61.2 mg, 86% yield. ^{1}H NMR (500 MHz, $CDCl_3$) δ 7.39 – 7.35 (m, 1H), 7.35 – 7.28 (m, 2H), 7.25 – 7.17 (m, 5H), 7.12 (d, J = 8.1 Hz, 2H), 6.88 (t, J = 7.3 Hz, 1H), 6.79 – 6.69 (m, 2H), 5.63 (s, 1H), 3.79 – 3.72 (m, 4H), 3.68 (ddd, J = 12.3, 10.2, 4.2 Hz, 1H), 3.14 (ddd, J = 16.2, 10.2, 6.0 Hz, 1H), 2.98 (dt, J = 16.1, 3.8 Hz, 1H). ^{13}C NMR (101 MHz, $CDCl_3$) δ 159.6, 149.8, 135.9, 134.6, 133.3, 129.3, 129.1, 127.6, 127.3, 126.4, 119.7, 116.9, 115.4, 113.9, 87.3, 84.8, 55.4, 52.5, 43.6, 29.1.

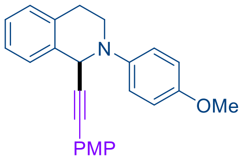

2-(4-Methoxyphenyl)-1-((4-methoxyphenyl)ethynyl)-1,2,3,4-tetrahydroisoquinoline[1] (**4**). Colorless oil, 63.2 mg, 81% yield. ^{1}H NMR (400 MHz, $CDCl_3$) δ 7.40 – 7.32 (m, 1H), 7.23 (q, *J* = 7.3, 6.0 Hz, 5H), 7.13 (d, *J* = 8.7 Hz, 2H), 6.96 – 6.84 (m, 2H), 6.76 (d, *J* = 8.3 Hz, 2H), 5.51 (s, 1H), 3.80 (s, 3H), 3.77 (s, 3H), 3.65 (td, *J* = 11.4, 4.1 Hz, 1H), 3.61 – 3.51 (m, 1H), 3.16 (ddd, *J* = 16.8, 10.7, 6.2 Hz, 1H), 2.94 (dt, *J* = 16.4, 3.4 Hz, 1H). ^{13}C NMR (101 MHz, $CDCl_3$) δ 159.5, 154.4, 144.4, 135.9, 134.2, 133.3, 129.2, 127.7, 127.2, 126.3, 120.3, 115.4, 114.6, 113.9, 87.2, 85.5, 55.8, 55.4, 54.6, 44.4, 29.2.

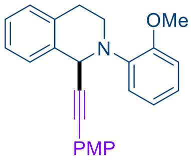

2-(2-Methoxyphenyl)-1-((4-methoxyphenyl)ethynyl)-1,2,3,4-tetrahydroisoquinoline[1] (**5**). Colorless oil, 53.2 mg, 69% yield. ^{1}H NMR (500 MHz, $CDCl_3$) δ 7.37 – 7.32 (m, 1H), 7.25 – 7.21 (m, 3H), 7.21 – 7.16 (m, 3H), 7.09 (td, *J* = 7.7, 1.6 Hz, 1H), 7.00 (td, *J* = 7.6, 1.5 Hz, 1H), 6.93 (dd, *J* = 8.1, 1.4 Hz, 1H), 6.77 – 6.72 (m, 2H), 5.76 (s, 1H), 3.89 (s, 3H), 3.76 (s, 3H), 3.71 (td, *J* = 11.7, 4.2 Hz, 1H), 3.46 (ddt, *J* = 11.9, 6.4, 1.5 Hz, 1H), 3.26 (ddd, *J* = 17.3, 11.5, 6.4 Hz, 1H), 2.92 (ddd, *J* = 16.4, 4.2, 1.7 Hz, 1H). ^{13}C NMR (126 MHz, $CDCl_3$) δ 159.4, 152.9, 140.0, 136.1, 133.9, 133.2, 129.3, 127.8, 127.0, 126.0, 123.7, 121.5, 120.9, 115.6, 113.8, 111.3, 87.5, 85.5, 55.7, 55.4, 53.1, 44.2, 29.4.

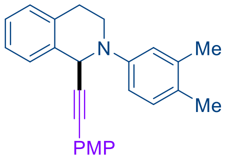

2-(3,4-Dimethylphenyl)-1-((4-methoxyphenyl)ethynyl)-1,2,3,4-tetrahydroisoquinoline[1] (**6**). Yellow oil, 42.3 mg, 55% yield. ^{1}H NMR (400 MHz, $CDCl_3$) δ 7.40 – 7.34 (m, 1H), 7.27 – 7.17 (m, 5H), 7.08 (d, *J* = 8.2 Hz, 1H), 6.96 (d, *J* = 2.6 Hz, 1H), 6.90 (dd, *J* = 8.2, 2.7 Hz, 1H), 6.79 – 6.73 (m, 2H), 5.59 (s, 1H), 3.77 (s, 3H), 3.74 – 3.61 (m, 2H), 3.15 (ddd, *J* = 16.5, 9.8, 6.9 Hz, 1H), 2.95 (dt, *J* = 16.2, 3.6 Hz, 1H), 2.29 (s, 3H), 2.22 (s, 3H). ^{13}C NMR (101 MHz, $CDCl_3$) δ

159.5, 148.1, 137.2, 136.0, 134.5, 133.3, 130.3, 129.1, 128.2, 127.6, 127.2, 126.3, 119.3, 115.5, 115.0, 113.9, 87.5, 85.1, 55.4, 53.1, 43.9, 29.2, 20.4, 19.0.

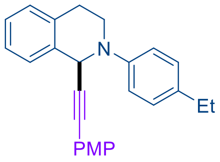

2-(4-Ethylphenyl)-1-((4-methoxyphenyl)ethynyl)-1,2,3,4-tetrahydroisoquinoline[1] (**7**). Yellow oil, 55.2 mg, 72% yield. ^{1}H NMR (500 MHz, $CDCl_3$) δ 7.41 – 7.35 (m, 1H), 7.28 – 7.15 (m, 7H), 7.09 (d, J = 8.5 Hz, 2H), 6.79 – 6.73 (m, 2H), 5.61 (s, 1H), 3.77 (s, 3H), 3.73 – 3.64 (m, 2H), 3.16 (ddd, J = 16.5, 10.0, 6.6 Hz, 1H), 2.97 (dt, J = 16.1, 3.7 Hz, 1H), 2.63 (q, J = 7.6 Hz, 2H), 1.25 (t, J = 7.6 Hz, 3H). ^{13}C NMR (126 MHz, $CDCl_3$) δ 159.5, 147.9, 135.9, 135.8, 134.5, 133.3, 129.1, 128.6, 127.6, 127.2, 126.3, 117.5, 115.4, 113.9, 87.4, 85.0, 55.4, 53.0, 43.9, 29.1, 28.2, 16.0.

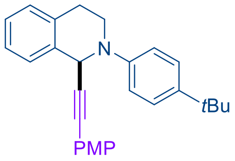

2-(4-(tert-Butyl)phenyl)-1-((4-methoxyphenyl)ethynyl)-1,2,3,4-tetrahydroisoquinoline[1] (**8**). Colorless oil, 55.3 mg, 67% yield. ^{1}H NMR (400 MHz, $CDCl_3$) δ 7.38 – 7.31 (m, 3H), 7.25 – 7.14 (m, 5H), 7.10 – 7.03 (m, 2H), 6.78 – 6.71 (m, 2H), 5.59 (s, 1H), 3.76 (s, 3H), 3.68 (tdd, J = 12.3, 8.2, 3.7 Hz, 2H), 3.13 (ddd, J = 16.2, 10.1, 6.2 Hz, 1H), 2.95 (dt, J = 16.1, 3.7 Hz, 1H), 1.31 (s, 9H). ^{13}C NMR (101 MHz, $CDCl_3$) δ 159.5, 147.4, 142.5, 136.0, 134.6, 133.4, 129.1, 127.6, 127.3, 126.4, 126.1, 116.7, 115.5, 113.9, 87.5, 84.9, 55.5, 52.7, 43.8, 34.2, 31.7, 29.2.

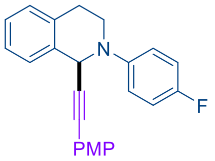

2-(4-Fluorophenyl)-1-((4-methoxyphenyl)ethynyl)-1,2,3,4-tetrahydroisoquinoline[1] **(9).** Yellow oil, 50.0 mg, 67% yield. ^{1}H NMR (500 MHz, $CDCl_3$) δ 7.36 (dt, J = 7.4, 3.7 Hz, 1H), 7.27 – 7.18 (m, 5H), 7.12 – 7.07 (m, 2H), 7.03 (t, J = 8.7 Hz, 2H), 6.79 – 6.74 (m, 2H), 5.54 (s, 1H), 3.76 (s, 3H), 3.63 (dddd, J = 21.1, 15.2, 11.3, 6.5 Hz, 2H), 3.15 (ddd, J = 16.5, 10.5, 6.2

Hz, 1H), 2.96 (dt, J = 16.2, 3.6 Hz, 1H). ^{13}C NMR (126 MHz, CDCl3) δ 159.5, 157.5 (d, *J* = 239.0 Hz), 146.6, 146.6, 135.5, 134.1, 133.2, 128.3 (d, *J* = 190.3 Hz), 126.8 (d, *J* = 121.2 Hz), 119.4 (d, *J* = 7.7 Hz), 115.7, 115.5, 115.1, 113.8, 86.8, 85.3, 55.3, 53.8, 44.1, 29.1. ^{19}F NMR (471 MHz, $CDCl_3$) δ -124.1.

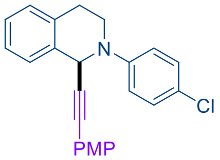

2-(4-Chlorophenyl)-1-((4-methoxyphenyl)ethynyl)-1,2,3,4-tetrahydroisoquinoline[1] (**10**). Yellow oil, 59.1 mg, 75% yield. ^{1}H NMR (500 MHz, $CDCl_3$) δ 7.35 (dd, *J* = 5.4, 3.6 Hz, 1H), 7.24 (dd, *J* = 5.2, 1.9 Hz, 4H), 7.22 (d, *J* = 2.7 Hz, 2H), 7.20 – 7.16 (m, 1H), 7.05 – 7.00 (m, 2H), 6.78 – 6.72 (m, 2H), 5.56 (s, 1H), 3.75 (s, 3H), 3.71 – 3.59 (m, 2H), 3.12 (ddd, *J* = 16.1, 9.8, 6.3 Hz, 1H), 2.97 (dt, *J* = 16.0, 3.9 Hz, 1H). ^{13}C NMR (126 MHz, $CDCl_3$) δ 159.6, 148.3, 135.4, 134.2, 133.3, 129.1, 129.0, 127.5, 127.4, 126.5, 124.5, 118.0, 115.0, 113.9, 86.7, 84.9, 55.4, 52.4, 43.7, 29.0.

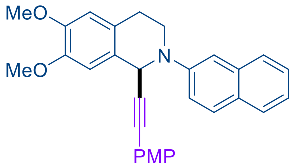

6,7-Dimethoxy-1-((4-methoxyphenyl)ethynyl)-2-(naphthalen-2-yl)-1,2,3,4-tetrahydroisoquinoline[1] (**11**). White solid, 60.9 mg, 64% yield. ^{1}H NMR (500 MHz, $CDCl_3$) δ 7.78 (d, *J* = 8.8 Hz, 1H), 7.73 (dd, *J* = 8.2, 3.4 Hz, 2H), 7.41 (ddd, *J* = 8.3, 4.9, 2.2 Hz, 3H), 7.32 – 7.27 (m, 1H), 7.25 – 7.20 (m, 2H), 6.91 (s, 1H), 6.76 – 6.70 (m, 2H), 6.68 (s, 1H), 5.70 (s, 1H), 3.95 – 3.86 (m, 7H), 3.79 – 3.70 (m, 4H), 3.13 (ddd, *J* = 16.6, 11.0, 6.0 Hz, 1H), 2.89 (dt, *J* = 15.8, 3.3 Hz, 1H). ^{13}C NMR (126 MHz, $CDCl_3$) δ 159.6, 148.5, 147.9, 147.6, 134.9, 133.4, 128.9, 128.7, 127.6, 127.6, 127.0, 126.5, 126.4, 123.5, 119.7, 115.3, 113.9, 111.8, 111.7, 110.5, 87.3, 85.1, 56.3, 56.2, 55.4, 52.4, 43.8, 28.7.

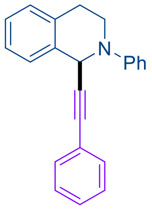

2-Phenyl-1-(phenylethynyl)-1,2,3,4-tetrahydroisoquinoline[2] (**12**). Colorless oil, 45.4 mg, 70%

yield. ^{1}H NMR (500 MHz, $CDCl_3$) δ 7.38 – 7.34 (m, 1H), 7.33 – 7.25 (m, 4H), 7.20 (tdd, *J* = 11.3, 5.6, 3.5 Hz, 6H), 7.11 (d, *J* = 8.1 Hz, 2H), 6.88 (t, *J* = 7.3 Hz, 1H), 5.63 (s, 1H), 3.74 (ddd, *J* = 12.7, 6.1, 3.5 Hz, 1H), 3.66 (ddd, *J* = 12.4, 10.3, 4.2 Hz, 1H), 3.13 (ddd, *J* = 16.3, 10.3, 6.0 Hz, 1H), 2.96 (dt, *J* = 16.0, 3.8 Hz, 1H). ^{13}C NMR (126 MHz, $CDCl_3$) δ 149.8, 135.6, 134.6, 131.9, 129.3, 129.1, 128.3, 128.2, 127.6, 127.4, 126.5, 123.2, 119.8, 116.9, 88.8, 85.0, 52.5, 43.6, 29.1.

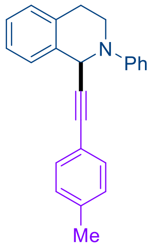

2-Phenyl-1-(p-tolylethynyl)-1,2,3,4-tetrahydroisoquinoline[2] (**13**). Colorless oil, 54.9 mg, 81% yield. ^{1}H NMR (500 MHz, $CDCl_3$) δ 7.34 – 7.29 (m, 1H), 7.29 – 7.23 (m, 2H), 7.20 – 7.16 (m, 2H), 7.16 – 7.11 (m, 3H), 7.09 – 7.04 (m, 2H), 6.96 (d, *J* = 7.9 Hz, 2H), 6.83 (tt, *J* = 7.3, 1.1 Hz, 1H), 5.58 (s, 1H), 3.72 – 3.66 (m, 1H), 3.62 (ddd, *J* = 12.3, 10.2, 4.2 Hz, 1H), 3.08 (ddd, *J* = 16.1, 10.2, 6.0 Hz, 1H), 2.92 (dt, *J* = 16.1, 3.8 Hz, 1H), 2.23 (s, 3H). ^{13}C NMR (126 MHz, $CDCl_3$) δ 149.8, 138.2, 135.8, 134.6, 131.8, 129.3, 129.1, 129.0, 127.6, 127.3, 126.4, 120.1, 119.7, 116.9, 88.0, 85.1, 52.5, 43.6, 29.1, 21.6.

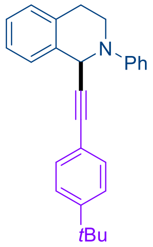

1-((4-(tert-Butyl)phenyl)ethynyl)-2-phenyl-1,2,3,4-tetrahydroisoquinoline[2] (**14**). Yellow oil, 60.1 mg, 78% yield. ^{1}H NMR (400 MHz, $CDCl_3$) δ 7.43 (dd, *J* = 5.3, 3.6 Hz, 1H), 7.40 – 7.34 (m, 2H), 7.28 (d, *J* = 10.4 Hz, 6H), 7.27 – 7.22 (m, 1H), 7.21 – 7.16 (m, 2H), 6.94 (tt, *J* = 7.2, 1.1 Hz, 1H), 5.70 (s, 1H), 3.80 (dddd, *J* = 12.5, 6.2, 3.5, 1.0 Hz, 1H), 3.73 (ddd, *J* = 12.3, 10.1, 4.2 Hz, 1H), 3.19 (ddd, *J* = 16.1, 10.1, 6.1 Hz, 1H), 3.03 (dt, *J* = 16.1, 3.8 Hz, 1H), 1.32 (s, 9H). ^{13}C NMR (101 MHz, $CDCl_3$) δ 151.4, 149.8, 135.7, 134.5, 131.6, 129.3, 129.1, 127.6, 127.3, 126.4, 125.2, 120.2, 119.8, 116.9, 88.1, 85.0, 52.6, 43.6, 34.9, 31.3, 29.1.

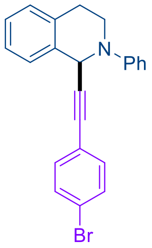

1-((4-Bromophenyl)ethynyl)-2-phenyl-1,2,3,4-tetrahydroisoquinoline[3] (**15**). Colorless oil, 58.0 mg, 71% yield. ^{1}H NMR (400 MHz, $CDCl_3$) δ 7.40 – 7.28 (m, 5H), 7.23 (ddd, *J* = 10.6, 6.4, 3.6 Hz, 3H), 7.18 – 7.07 (m, 4H), 6.90 (tt, *J* = 7.2, 1.1 Hz, 1H), 5.63 (s, 1H), 3.76 (dddd, *J* = 12.3, 6.0, 3.5, 1.1 Hz, 1H), 3.65 (ddd, *J* = 12.3, 10.2, 4.2 Hz, 1H), 3.15 (ddd, *J* = 16.2, 10.2, 5.9 Hz, 1H), 2.98 (dt, *J* = 16.1, 3.9 Hz, 1H). ^{13}C NMR (101 MHz, $CDCl_3$) δ 149.7, 135.3, 134.6, 133.4, 131.5, 129.4, 129.2, 127.6, 127.5, 126.5, 122.4, 122.1, 120.0, 116.9, 90.0, 83.9, 52.6, 43.6, 29.1.

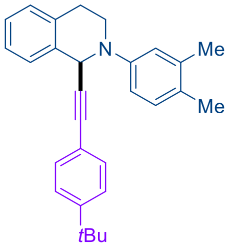

1-((4-(tert-Butyl)phenyl)ethynyl)-2-(3,4-dimethylphenyl)-1,2,3,4-tetrahydroisoquinoline[1] (**16**). Yellow oil, 56.4 mg, 68% yield. ^{1}H NMR (400 MHz, $CDCl_3$) δ 7.42 – 7.36 (m, 1H), 7.31 – 7.18 (m, 7H), 7.10 (d, *J* = 8.2 Hz, 1H), 6.99 (d, *J* = 2.6 Hz, 1H), 6.93 (dd, *J* = 8.2, 2.7 Hz, 1H), 5.62 (s, 1H), 3.76 – 3.63 (m, 2H), 3.17 (ddd, *J* = 16.5, 9.8, 6.8 Hz, 1H), 2.97 (dt, *J* = 16.2, 3.6 Hz, 1H), 2.31 (s, 3H), 2.24 (s, 3H), 1.30 (s, 9H). ^{13}C NMR (101 MHz, $CDCl_3$) δ 151.3, 148.1, 137.2, 135.9, 134.5, 131.6, 130.3, 129.1, 128.3, 127.6, 127.2, 126.3, 125.2, 120.3, 119.3, 115.1, 88.3, 85.4, 53.2, 43.9, 34.9, 31.3, 29.2, 20.4, 19.0.

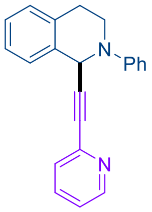

2-Phenyl-1-(pyridin-2-ylethynyl)-1,2,3,4-tetrahydroisoquinoline[4] (**17**). Colorless oil, 46.6 mg, 72% yield. ^{1}H NMR (400 MHz, $CDCl_3$) δ 8.54 – 8.46 (m, 1H), 7.54 (td, *J* = 7.8, 1.8 Hz, 1H), 7.42 – 7.38 (m, 1H), 7.36 – 7.29 (m, 2H), 7.29 – 7.26 (m, 1H), 7.21 (dtd, *J* = 9.4, 5.6, 3.7

Hz, 3H), 7.14 (dd, J = 7.9, 5.3 Hz, 3H), 6.89 (t, J = 7.3 Hz, 1H), 5.69 (s, 1H), 3.79 (ddd, J = 12.5, 6.0, 3.6 Hz, 1H), 3.72 (ddd, J = 12.4, 10.0, 4.2 Hz, 1H), 3.16 (ddd, J = 16.1, 10.0, 6.1 Hz, 1H), 3.00 (dt, J = 16.1, 3.9 Hz, 1H). ^{13}C NMR (101 MHz, $CDCl_3$) δ 150.0, 149.5, 143.3, 136.1, 134.8, 134.7, 129.4, 129.1, 127.7, 127.6, 127.5, 126.6, 122.8, 119.8, 116.6, 88.9, 84.4, 52.2, 43.7, 29.0.

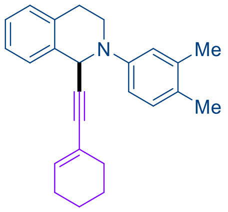

1-(Cyclohex-1-en-1-ylethynyl)-2-(3,4-dimethylphenyl)-1,2,3,4-tetrahydroisoquinoline[1] (**18**). Colorless oil, 54.1 mg, 76% yield. ^{1}H NMR (400 MHz, $CDCl_3$) δ 7.32 (dd, J = 5.5, 3.6 Hz, 1H), 7.25 – 7.15 (m, 3H), 7.08 (d, J = 8.2 Hz, 1H), 6.93 (d, J = 2.6 Hz, 1H), 6.87 (dd, J = 8.2, 2.7 Hz, 1H), 6.05 – 5.92 (m, 1H), 5.50 (s, 1H), 3.72 – 3.56 (m, 2H), 3.13 (ddd, J = 16.5, 10.3, 6.4 Hz, 1H), 2.93 (dt, J = 16.1, 3.6 Hz, 1H), 2.29 (s, 3H), 2.23 (s, 3H), 2.03 (p, J = 4.2, 3.4 Hz, 4H), 1.64 – 1.49 (m, 4H). ^{13}C NMR (101 MHz, $CDCl_3$) δ 148.1, 137.1, 136.3, 134.5, 134.4, 130.2, 129.1, 128.0, 127.6, 127.1, 126.2, 120.7, 119.2, 114.9, 87.1, 86.0, 52.9, 43.8, 29.5, 29.1, 25.7, 22.4, 21.7, 20.4, 19.0.

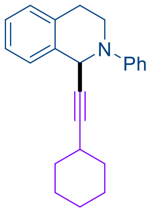

1-(Cyclohexylethynyl)-2-phenyl-1,2,3,4-tetrahydroisoquinoline[2] (**19**). Yellow oil, 41.5 mg, 63% yield. ^{1}H NMR (500 MHz, $CDCl_3$) δ 7.31 (ddd, J = 8.9, 6.1, 3.3 Hz, 3H), 7.24 – 7.15 (m, 3H), 7.09 (d, J = 8.1 Hz, 2H), 6.88 (t, J = 7.3 Hz, 1H), 5.45 (s, 1H), 3.71 (ddd, J = 12.4, 5.9, 3.4 Hz, 1H), 3.59 (ddd, J = 12.3, 10.5, 4.2 Hz, 1H), 3.11 (ddd, J = 16.3, 10.5, 5.9 Hz, 1H), 2.94 (dt, J = 16.0, 3.8 Hz, 1H), 2.39 – 2.26 (m, 1H), 1.65 (ddd, J = 11.6, 7.3, 3.5 Hz, 2H), 1.57 (dtt, J = 13.5, 6.9, 3.4 Hz, 2H), 1.38 – 1.17 (m, 6H). ^{13}C NMR (126 MHz, $CDCl_3$) δ 149.9, 136.5, 134.3, 129.1, 128.9, 127.5, 127.0, 126.2, 119.5, 116.9, 89.5, 79.3, 52.1, 43.3, 32.8, 32.7, 29.1, 29.1, 26.1, 24.7.

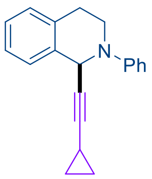

1-(Cyclopropylethynyl)-2-phenyl-1,2,3,4-tetrahydroisoquinoline[2] (**20**). Colorless oil, 32.9 mg, 57% yield. ^{1}H NMR (500 MHz, $CDCl_3$) δ 7.31 (dt, J = 9.1, 6.3 Hz, 3H), 7.21 (dt, J = 7.3, 3.8 Hz, 2H), 7.19 – 7.15 (m, 1H), 7.06 (d, J = 8.0 Hz, 2H), 6.90 – 6.85 (m, 1H), 5.40 (s, 1H), 3.70 (ddt, J = 10.5, 3.9, 2.3 Hz, 1H), 3.60 (ddd, J = 12.3, 10.4, 4.2 Hz, 1H), 3.10 (ddd, J = 16.2, 10.4, 5.9 Hz, 1H), 2.94 (dt, J = 16.1, 3.9 Hz, 1H), 1.16 (dddd, J = 13.2, 8.2, 4.9, 1.6 Hz, 1H), 0.67 (dt, J = 8.2, 3.1 Hz, 2H), 0.55 (dt, J = 4.7, 3.1 Hz, 2H). ^{13}C NMR (126 MHz, $CDCl_3$) δ 149.8, 136.3, 134.4, 129.2, 129.0, 127.5, 127.2, 126.3, 119.5, 116.7, 88.5, 74.5, 51.9, 43.4, 29.0, 8.5, -0.2.

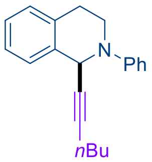

1-(Hex-1-yn-1-yl)-2-phenyl-1,2,3,4-tetrahydroisoquinoline[2] (**21**). Yellow oil, 24.1 mg, 40% yield. ^{1}H NMR (400 MHz, $CDCl_3$) δ 7.31 (td, J = 7.2, 3.2 Hz, 3H), 7.20 (ddd, J = 13.2, 7.8, 3.8 Hz, 3H), 7.12 – 7.04 (m, 2H), 6.88 (t, J = 7.3 Hz, 1H), 5.43 (s, 1H), 3.71 (ddd, J = 12.4, 6.0, 3.6 Hz, 1H), 3.60 (ddd, J = 12.2, 10.3, 4.2 Hz, 1H), 3.11 (ddd, J = 16.2, 10.3, 6.0 Hz, 1H), 2.94 (dt, J = 16.1, 3.9 Hz, 1H), 2.11 (td, J = 7.0, 2.0 Hz, 2H), 1.43 – 1.34 (m, 2H), 1.33 – 1.23 (m, 2H), 0.84 (t, J = 7.2 Hz, 3H). ^{13}C NMR (101 MHz, $CDCl_3$) δ 149.9, 136.4, 134.4, 129.2, 129.0, 127.5, 127.1, 126.3, 119.5, 116.8, 85.4, 79.3, 52.0, 43.4, 31.0, 29.1, 22.0, 18.7, 13.7.

5. Synthesis and characterization for the 2-aryl-1,2,3,4-tetrahydroisoquinolines

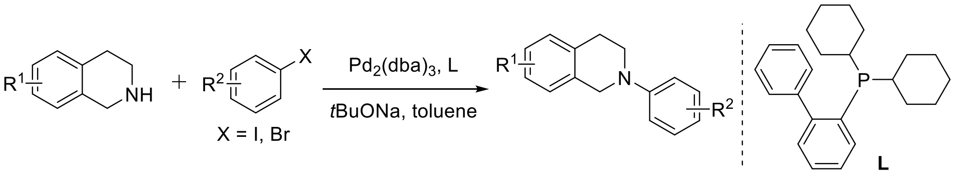

Scheme S1. General reaction for synthesizing of 2-aryl-1,2,3,4-tetrahydroisoquinolines.

General Procedure: The substrates were prepared by following a modified reported procedure.[5] A mixture of $Pd_2(dba)_3$ (3 mol %), ligand (2-(dicyclohexylphosphino)biphenyl) (8 mol %) and 15 mL of dry toluene were placed in an oven-dried tube. The tube was sealed and the solution was degassed with bubbling argon for 1 h. Aryl halide (1.0 equiv), 1,2,3,4-tetrahydroisoquinolines (1.2 equiv), and *t*-BuONa (1.4 equiv) were added sequentially. Then the reaction mixture was heated at 100 °C for 12 h. The reaction mixture was cooled to room temperature, quenched by adding water, and extracted with ethyl acetate. The organic layer was dried over anhydrous Na_2SO_4 and concentrated under reduced pressure. The crude product was purified by column chromatography on silica gel using hexanes/ethyl acetate as the eluent to afford the desired products.

Note: all the substrates are known in the literature.

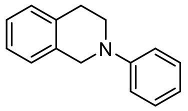

2-Phenyl-1,2,3,4-tetrahydroisoquinoline[1] (**1a**) was synthesized following **General Procedure** from 1,2,3,4-tetrahydroisoquinoline and iodobenzene. Colorless oil, 60% yield. ^{1}H NMR (400 MHz, $CDCl_3$) δ 7.37 – 7.29 (m, 2H), 7.26 – 7.14 (m, 4H), 7.08 – 6.96 (m, 2H), 6.87 (tt, *J* = 7.3, 1.1 Hz, 1H), 4.45 (s, 2H), 3.60 (t, *J* = 5.9 Hz, 2H), 3.02 (t, *J* = 5.8 Hz, 2H). ^{13}C NMR (101 MHz, $CDCl_3$) δ 150.8, 135.1, 134.7, 129.4, 128.7, 126.7, 126.5, 126.2, 118.9, 115.3, 51.0, 46.7, 29.3.

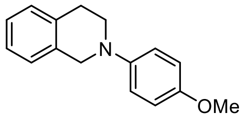

2-(4-Methoxyphenyl)-1,2,3,4-tetrahydroisoquinoline[1] (**1b**) was synthesized following **General Procedure** from 1,2,3,4-tetrahydroisoquinoline and 1-iodo-4-methoxybenzene. White solid, 41% yield. ^{1}H NMR (500 MHz, $CDCl_3$) δ 7.20 – 7.10 (m, 4H), 7.01 – 6.95 (m, 2H), 6.90 – 6.84 (m, 2H), 4.30 (s, 2H), 3.78 (s, 3H), 3.45 (t, *J* = 5.9 Hz, 2H), 2.99 (t, *J* = 5.9 Hz, 2H). ^{13}C NMR (126 MHz, $CDCl_3$) δ 153.7, 145.6, 134.8, 134.8, 128.9, 126.7, 126.5, 126.1, 118.2, 114.8, 55.9, 52.9, 48.7, 29.3.

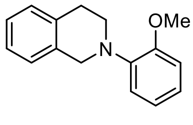

2-(2-Methoxyphenyl)-1,2,3,4-tetrahydroisoquinoline[1] (**1c**) was synthesized following **General Procedure** from 1,2,3,4-tetrahydroisoquinoline and 1-iodo-2-methoxybenzene. Yellow oil, 33% yield. ^{1}H NMR (500 MHz, $CDCl_3$) δ 7.19 – 7.12 (m, 3H), 7.12 – 7.08 (m, 1H), 7.02 (dd, J = 8.0, 6.7 Hz, 2H), 6.96 – 6.87 (m, 2H), 4.30 (s, 2H), 3.90 (s, 3H), 3.42 (t, J = 5.8 Hz, 2H), 2.99 (t, J = 5.8 Hz, 2H). ^{13}C NMR (126 MHz, $CDCl_3$) δ 152.8, 141.4, 135.4, 134.8, 129.1, 126.6, 126.3, 125.9, 123.2, 121.1, 119.2, 111.5, 55.7, 53.3, 49.2, 29.1.

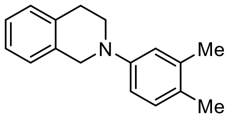

2-(3,4-Dimethylphenyl)-1,2,3,4-tetrahydroisoquinoline[1] (**1d**) was synthesized following **General Procedure** from 1,2,3,4-tetrahydroisoquinoline and 4-bromo-1,2-dimethylbenzene. White solid, 67% yield. ^{1}H NMR (500 MHz, $CDCl_3$) δ 7.18 (tdd, J = 9.3, 6.1, 3.5 Hz, 4H), 7.06 (d, J = 8.2 Hz, 1H), 6.84 (d, J = 2.6 Hz, 1H), 6.78 (dd, J = 8.2, 2.7 Hz, 1H), 4.37 (s, 2H), 3.52 (t, J = 5.8 Hz, 2H), 3.00 (t, J = 5.8 Hz, 2H), 2.28 (s, 3H), 2.21 (s, 3H). ^{13}C NMR (126 MHz, $CDCl_3$) δ 149.3, 137.4, 135.0, 134.9, 130.4, 128.8, 127.4, 126.7, 126.4, 126.1, 117.7, 113.5, 51.8, 47.5, 29.4, 20.5, 18.9.

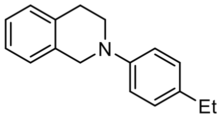

2-(4-Ethylphenyl)-1,2,3,4-tetrahydroisoquinoline1 (1e) was synthesized following **General Procedure** from 1,2,3,4-tetrahydroisoquinoline and 1-ethyl-4-iodobenzene. White solid, 79% yield. ^{1}H NMR (500 MHz, $CDCl_3$) δ 7.23 – 7.09 (m, 6H), 6.98 – 6.92 (m, 2H), 4.38 (s, 2H), 3.53 (t, J = 5.8 Hz, 2H), 3.00 (t, J = 5.8 Hz, 2H), 2.60 (q, J = 7.6 Hz, 2H), 1.23 (t, J = 7.6 Hz, 3H). ^{13}C NMR (126 MHz, $CDCl_3$) δ 149.0, 135.1, 135.0, 134.8, 128.8, 128.7, 126.7, 126.5, 126.1, 116.0, 51.6, 47.4, 29.4, 28.1, 16.0.

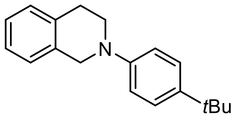

2-(4-(*tert*-Butyl)phenyl)-1,2,3,4-tetrahydroisoquinoline[1] (**1f**) was synthesized following **General Procedure** from 1,2,3,4-tetrahydroisoquinoline and 1-(*tert*-butyl)-4-iodobenzene. Light yellow solid, 74% yield. ^{1}H NMR (500 MHz, $CDCl_3$) δ 7.34 – 7.29 (m, 2H), 7.17 (qq, J = 6.3, 3.5, 2.8 Hz, 4H), 6.98 – 6.92 (m, 2H), 4.39 (s, 2H), 3.54 (t, J = 5.8 Hz, 2H), 2.99 (t, J = 5.8 Hz, 2H), 1.31 (s,

9H). ^{13}C NMR (126 MHz, $CDCl_3$) δ 148.6, 141.8, 135.1, 134.8, 128.8, 126.7, 126.5, 126.2, 126.2, 115.3, 51.4, 47.0, 34.1, 31.7, 29.4.

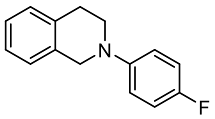

2-(4-Fluorophenyl)-1,2,3,4-tetrahydroisoquinoline[1] (**1g**) was synthesized following **General Procedure** from 1,2,3,4-tetrahydroisoquinoline and 1-fluoro-4-iodobenzene. White solid, 74% yield. ^{1}H NMR (500 MHz, $CDCl_3$) δ 7.18 (ddt, J = 17.2, 11.9, 4.7 Hz, 4H), 7.00 (t, J = 8.7 Hz, 2H), 6.97 – 6.91 (m, 2H), 4.34 (s, 2H), 3.50 (t, J = 5.9 Hz, 2H), 3.00 (t, J = 5.9 Hz, 2H). ^{13}C NMR (126 MHz, $CDCl_3$) δ 157.0 (d, J = 237.9 Hz), 147.6 (d, J = 2.1 Hz), 134.6 (d, J = 31.2 Hz), 127.8 (d, J = 267.7 Hz), 126.6, 126.2, 117.4, 117.3, 115.9, 115.7, 52.1, 48.0, 29.2. ^{19}F NMR (471 MHz, $CDCl_3$) δ -125.6.

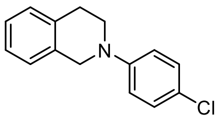

2-(4-Chlorophenyl)-1,2,3,4-tetrahydroisoquinoline[1] (**1h**) was synthesized following **General Procedure** from 1,2,3,4-tetrahydroisoquinoline and 1-chloro-4-iodobenzene. White solid, 56% yield. ^{1}H NMR (400 MHz, $CDCl_3$) δ 7.25 – 7.11 (m, 6H), 6.92 – 6.85 (m, 2H), 4.38 (s, 2H), 3.54 (t, J = 5.9 Hz, 2H), 2.98 (t, J = 5.8 Hz, 2H). ^{13}C NMR (101 MHz, $CDCl_3$) δ 149.3, 134.9, 134.3, 129.2, 128.7, 126.7, 126.7, 126.4, 123.5, 116.3, 50.9, 46.7, 29.2**.**

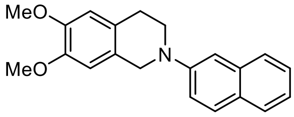

6,7-Dimethoxy-2-(naphthalen-2-yl)-1,2,3,4-tetrahydroisoquinoline[1] (**1i**) was synthesized following **General Procedure** from 6,7-dimethoxy-1,2,3,4-tetrahydroisoquinoline and 2-bromonaphthalene. White solid, 44% yield. ^{1}H NMR (500 MHz, $CDCl_3$) δ 7.75 (d, J = 9.0 Hz, 1H), 7.70 (dd, J = 14.4, 8.1 Hz, 2H), 7.39 (ddd, J = 8.1, 6.8, 1.3 Hz, 1H), 7.35 (dd, J = 9.0, 2.5 Hz, 1H), 7.29 – 7.24 (m, 2H), 7.17 (d, J = 2.4 Hz, 1H), 6.69 (d, J = 22.9 Hz, 2H), 4.44 (s, 2H), 3.90 (s, 3H), 3.88 (s, 3H), 3.66 (t, J = 5.8 Hz, 2H), 2.96 (t, J = 5.8 Hz, 2H). ^{13}C NMR (126 MHz, $CDCl_3$) δ 148.7, 147.9, 147.8, 135.0, 129.0, 128.3, 127.7, 126.8, 126.8, 126.5, 126.4, 123.2, 119.1, 111.7, 109.7, 109.7, 56.2, 56.2, 51.1, 47.6, 28.9.

6. Reference

(1) Gao, P.-S.; Weng, X.-J.; Wang, Z.-H.; Zheng, C.; Sun, B.; Chen, Z.-H.; You, S.-L.; Mei, T.-S., *Angew. Chem. Int. Ed.* **2020,** 59, 15254-15259.

(2) Rueping, M.; Koenigs, R. M.; Poscharny, K.; Fabry, D. C.; Leonori, D.; Vila, C., *Chem. Eur. J.* **2012,** 18, 5170-5174.

(3) Li, Z. P.; Li, C. J., *Org. Lett.* **2004,** 6, 4997-4999.

(4) Su, W.; Yu, J.; Li, Z.; Jiang, Z., *J. Org. Chem.* **2011,** 76, 9144-9150.

(5) Mudithanapelli, C.; Dhorma, L. P.; Kim, M.-h., *Org. Lett.* **2019,** 21, 3098-3102.

7. NMR spectra

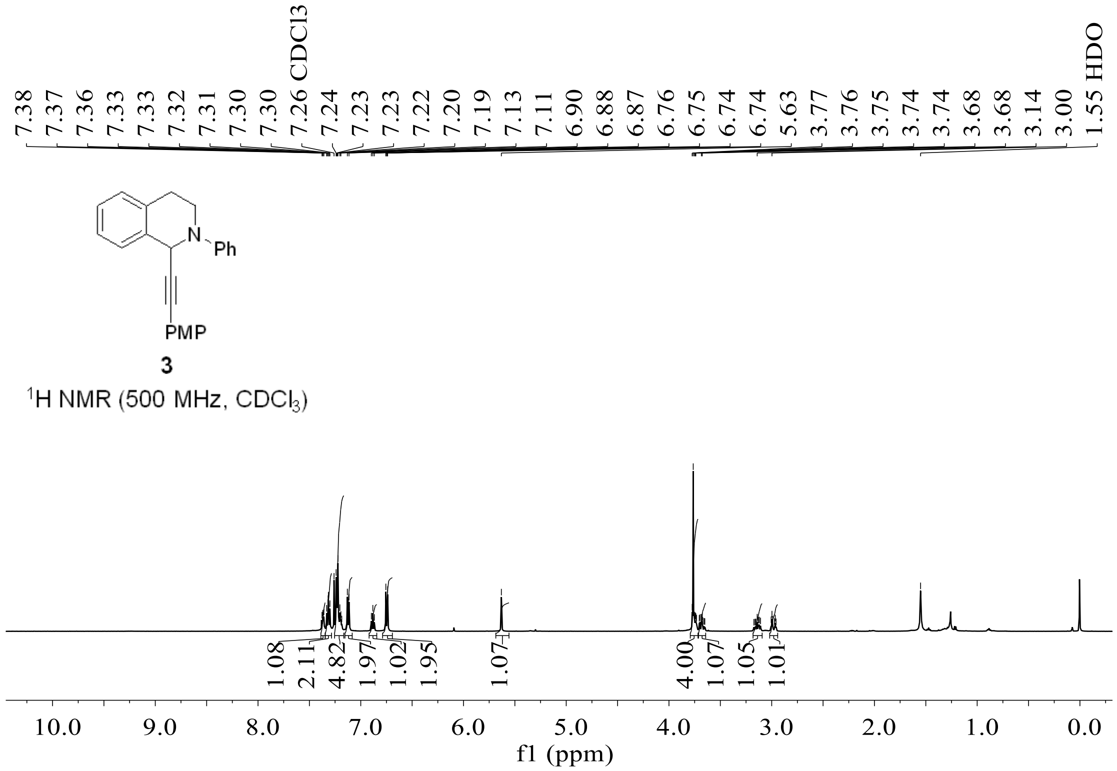

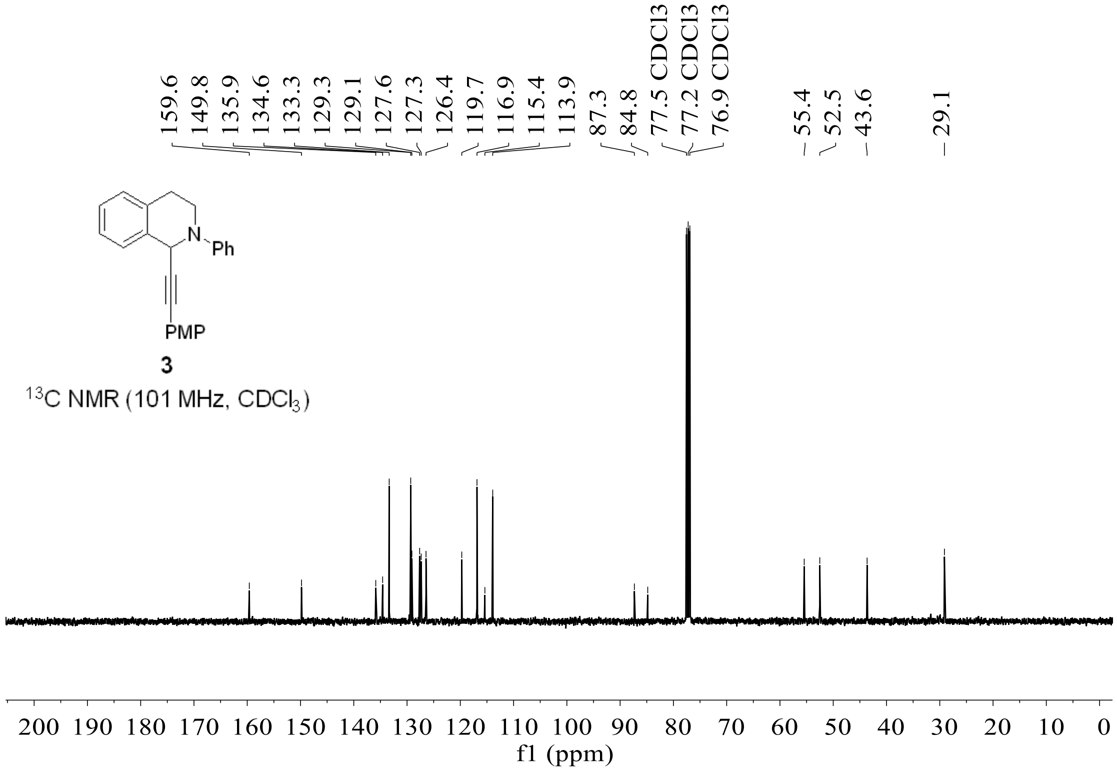

Figure S2. ^{1}H NMR and ^{13}C NMR spectra of compound **3**.

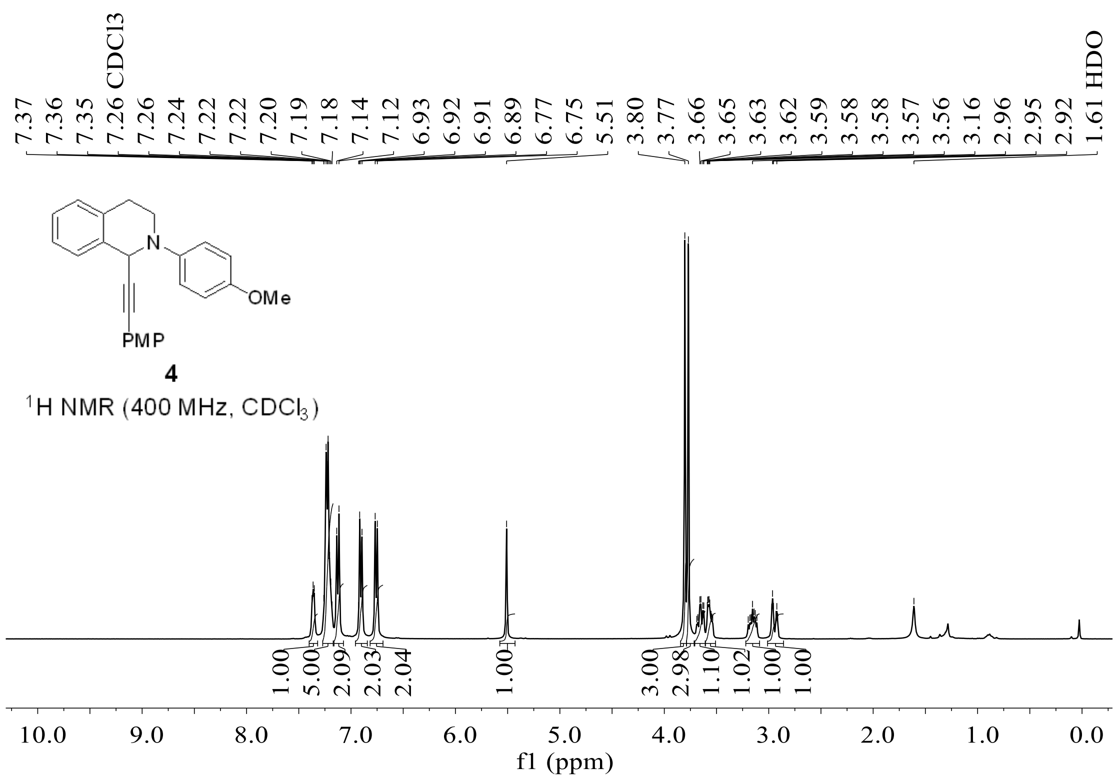

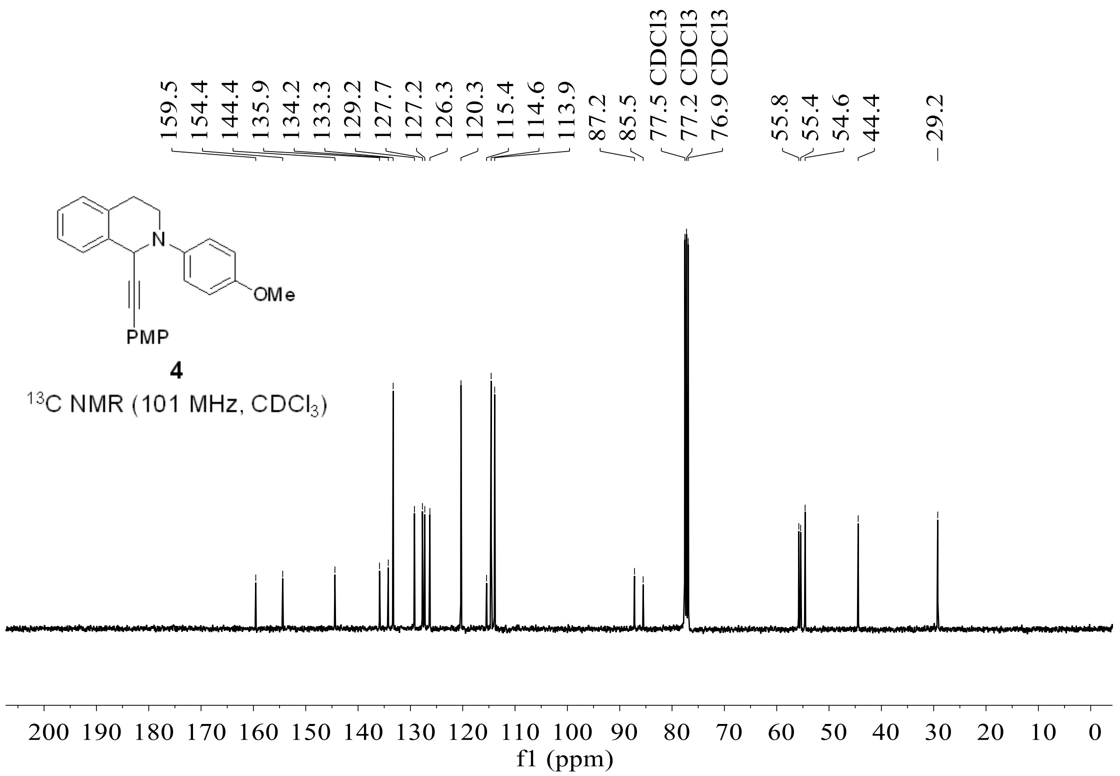

Figure S3. ^{1}H NMR and ^{13}C NMR spectra of compound **4**.

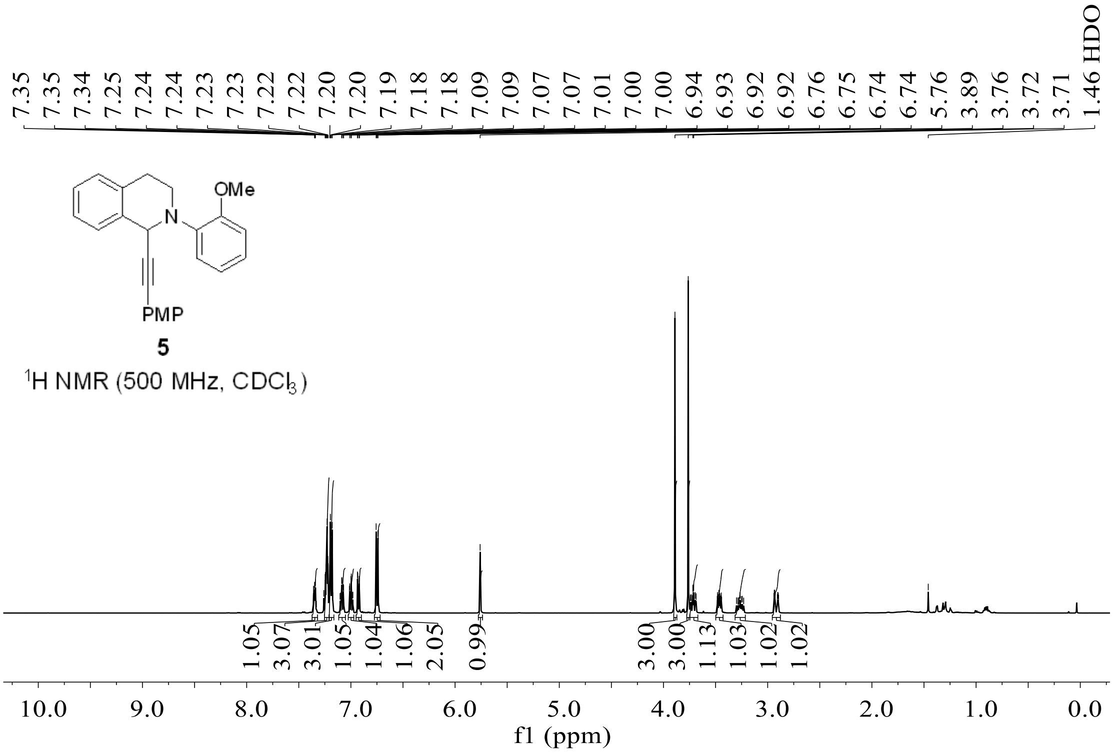

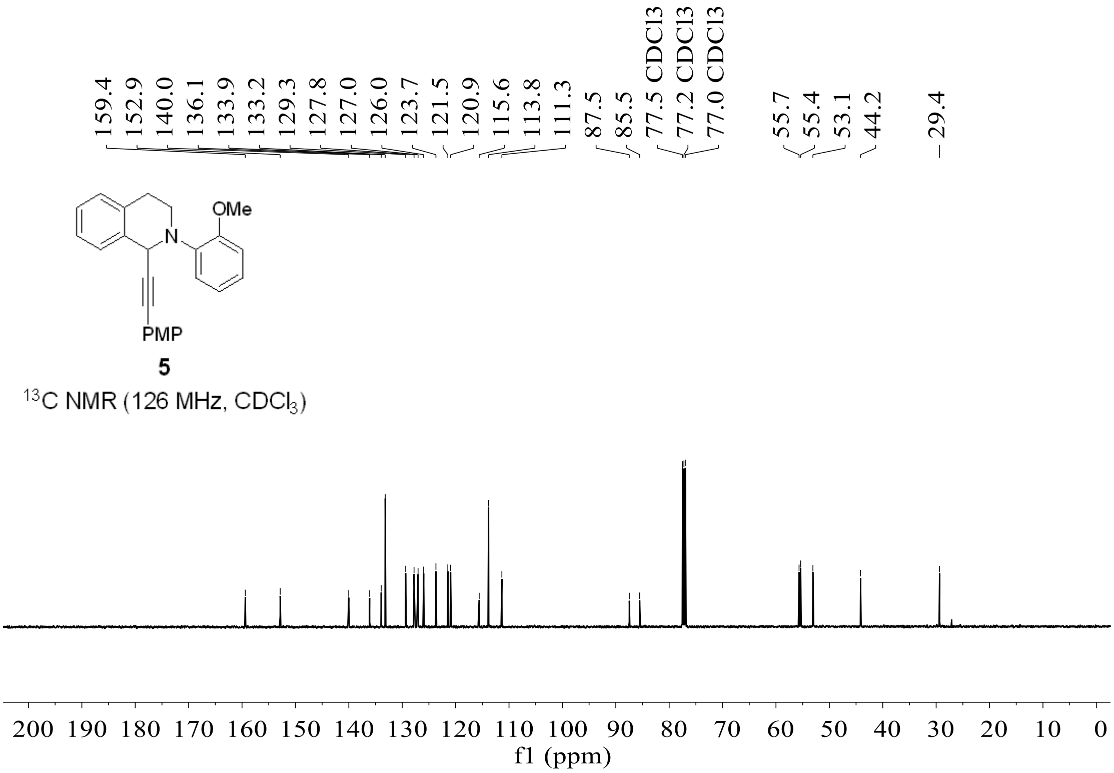

Figure S4. ^{1}H NMR and ^{13}C NMR spectra of compound **5**.

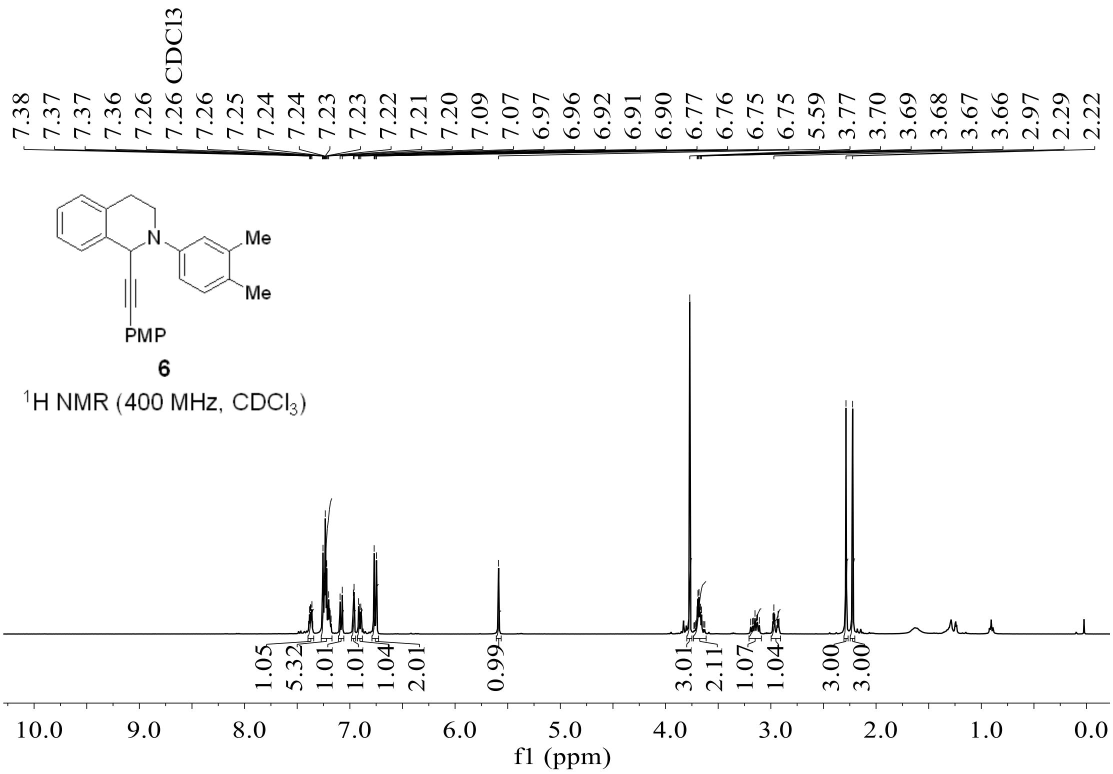

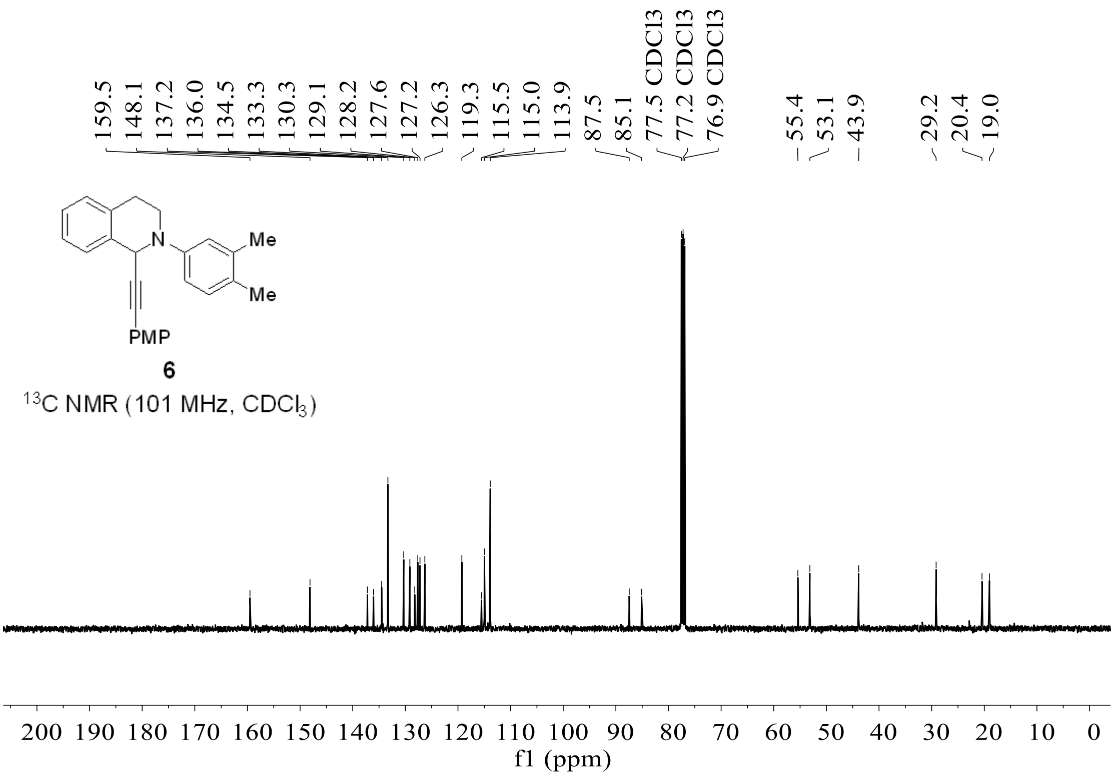

Figure S5. ^{1}H NMR and ^{13}C NMR spectra of compound **6**.

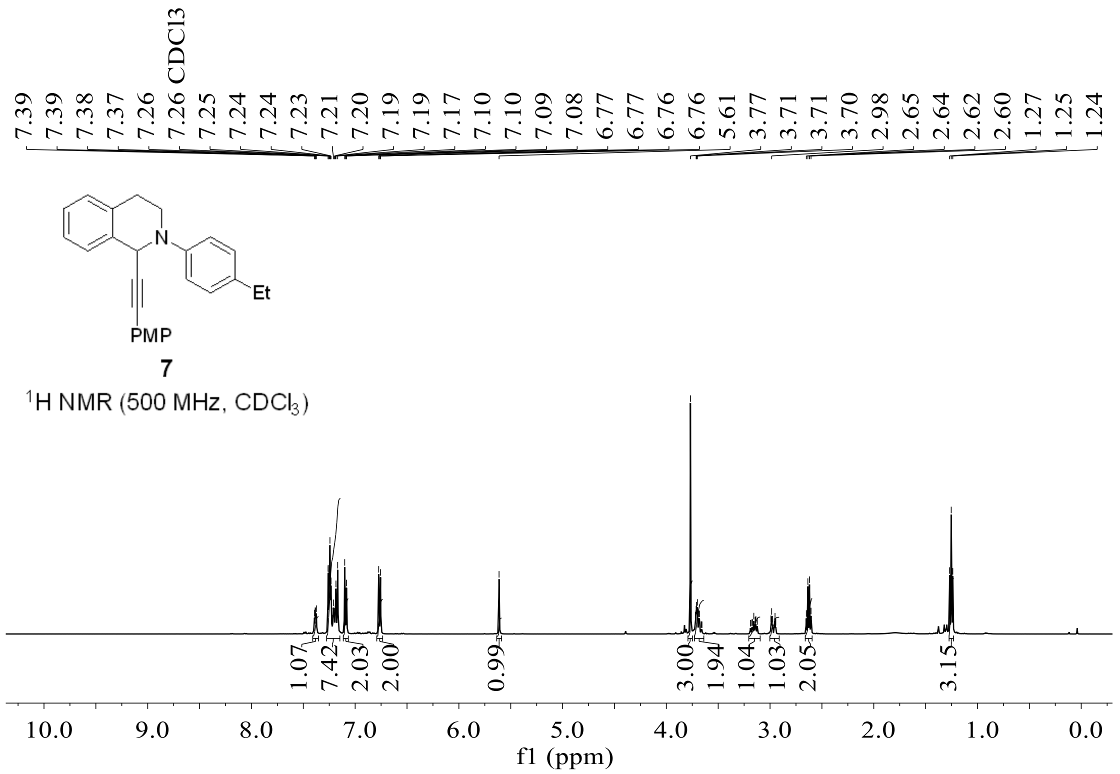

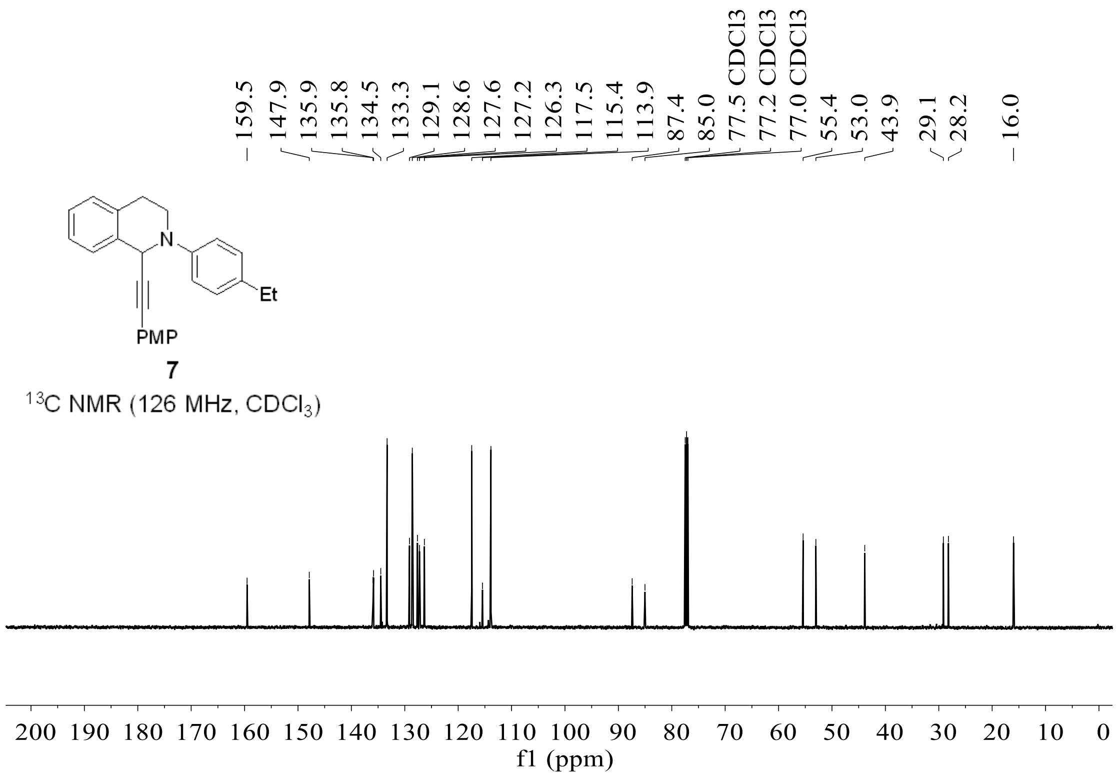

Figure S6. ^{1}H NMR and ^{13}C NMR spectra of compound **7**.

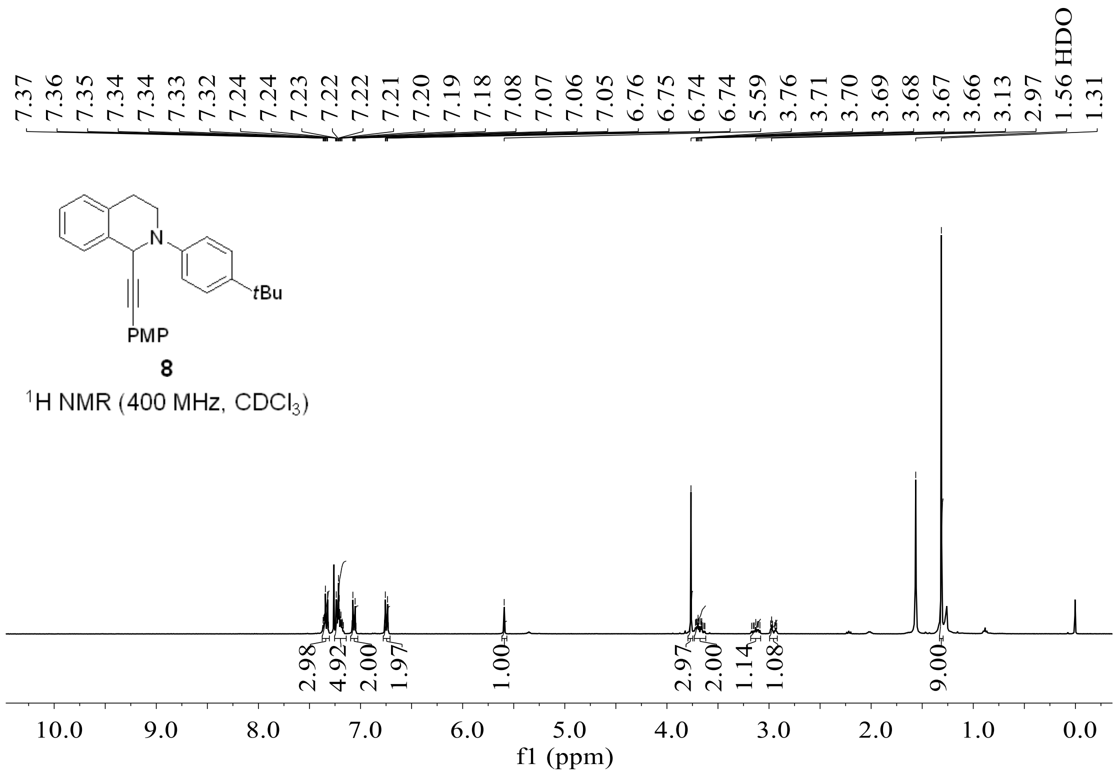

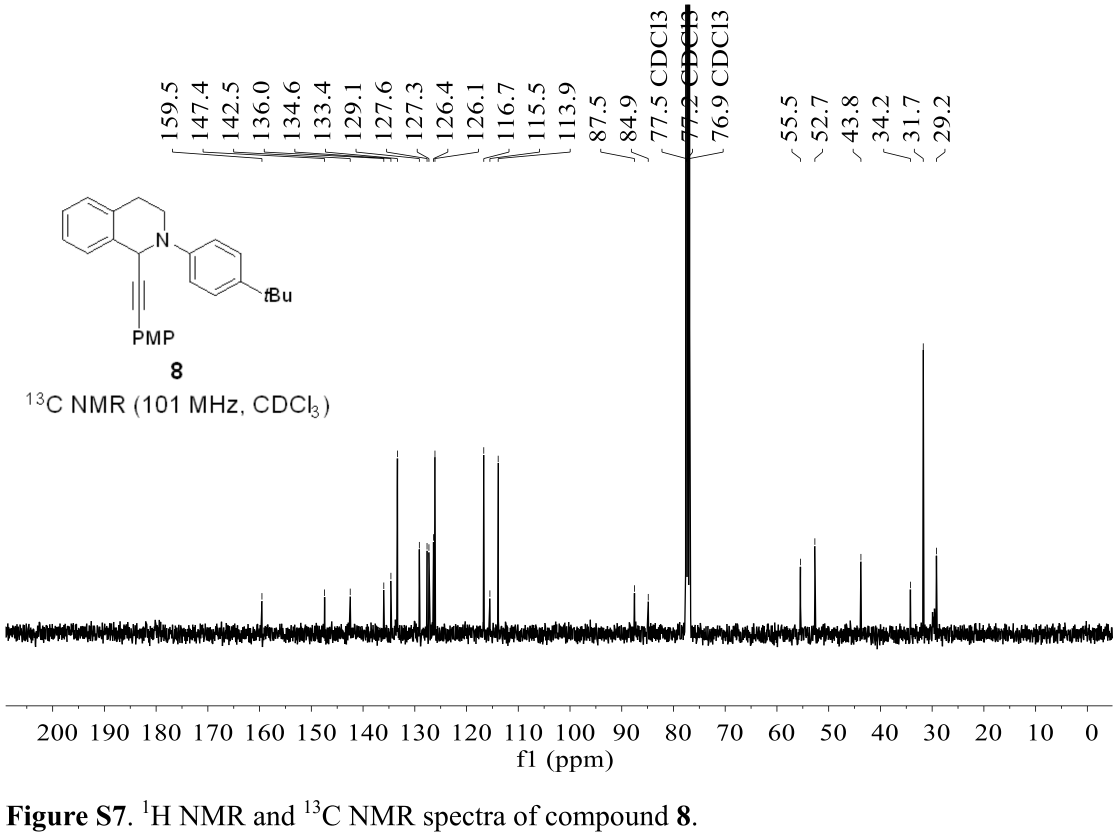

Figure S7. ^{1}H NMR and ^{13}C NMR spectra of compound **8**.

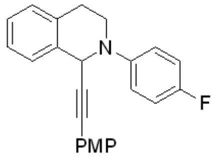

9

^{1}H NMR (500 MHz, $CDCl_3$)

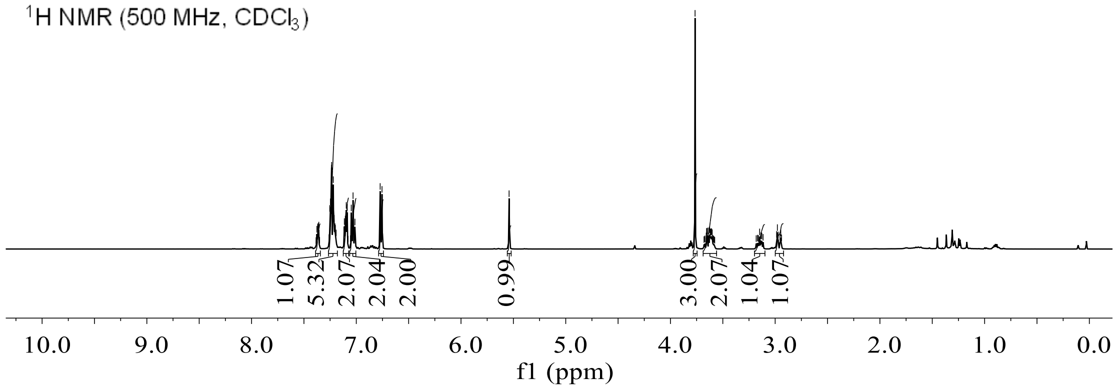

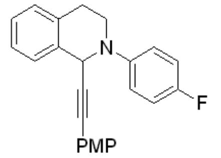

9

^{13}C NMR (126 MHz, $CDCl_3$)

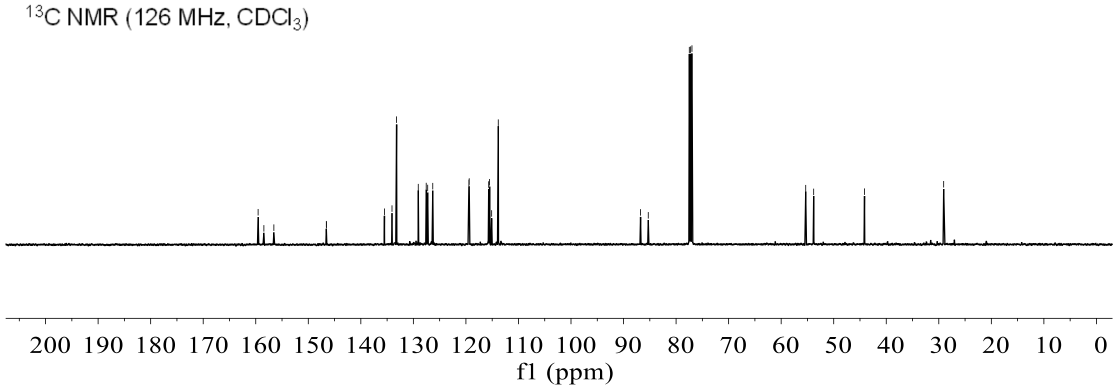

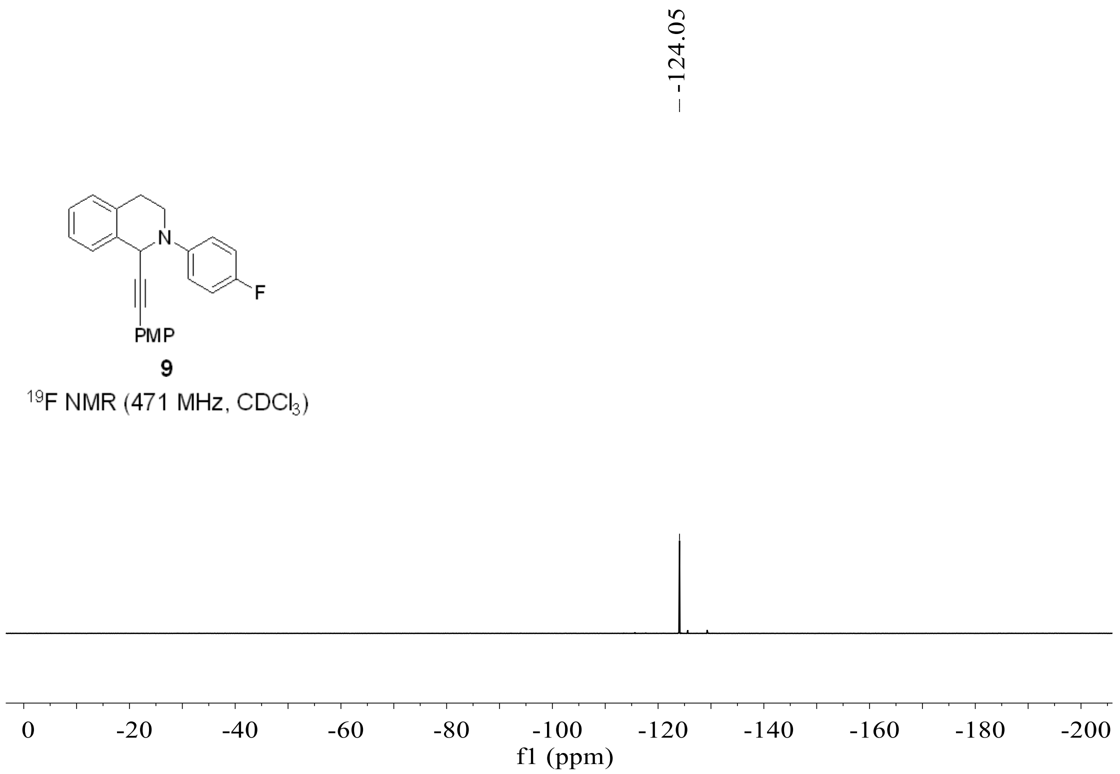

Figure S8. ^{1}H NMR, ^{13}C NMR and ^{19}F NMR spectra of compound **9**.

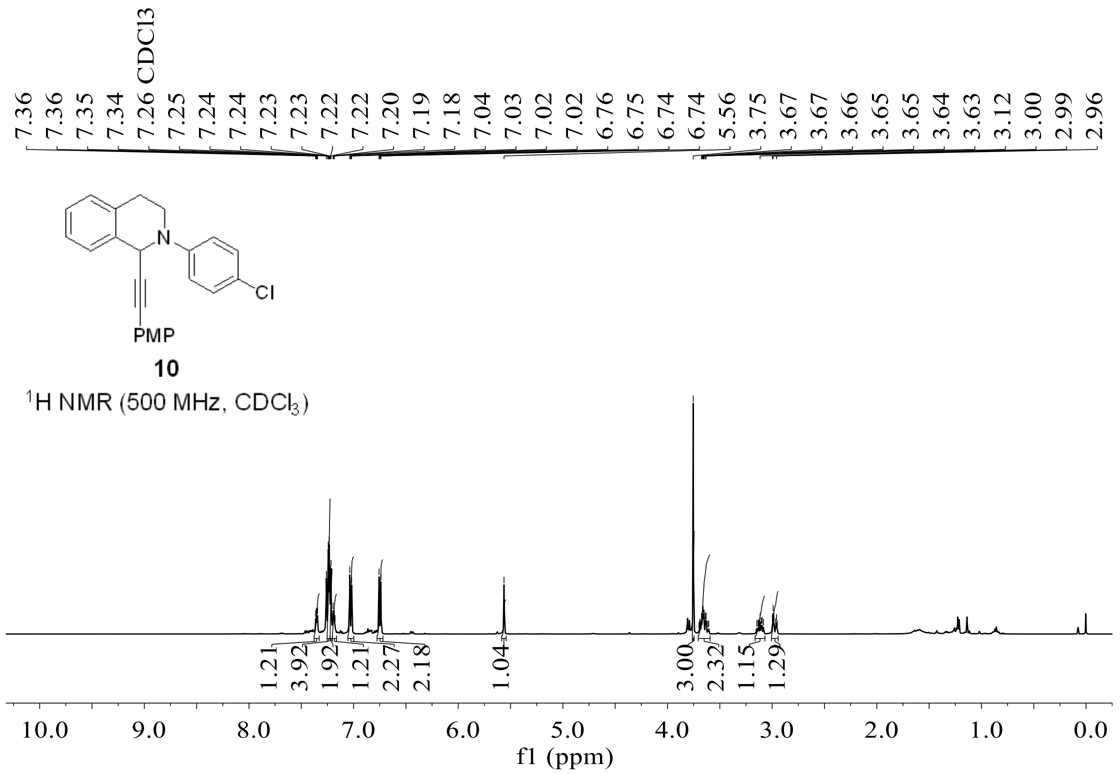

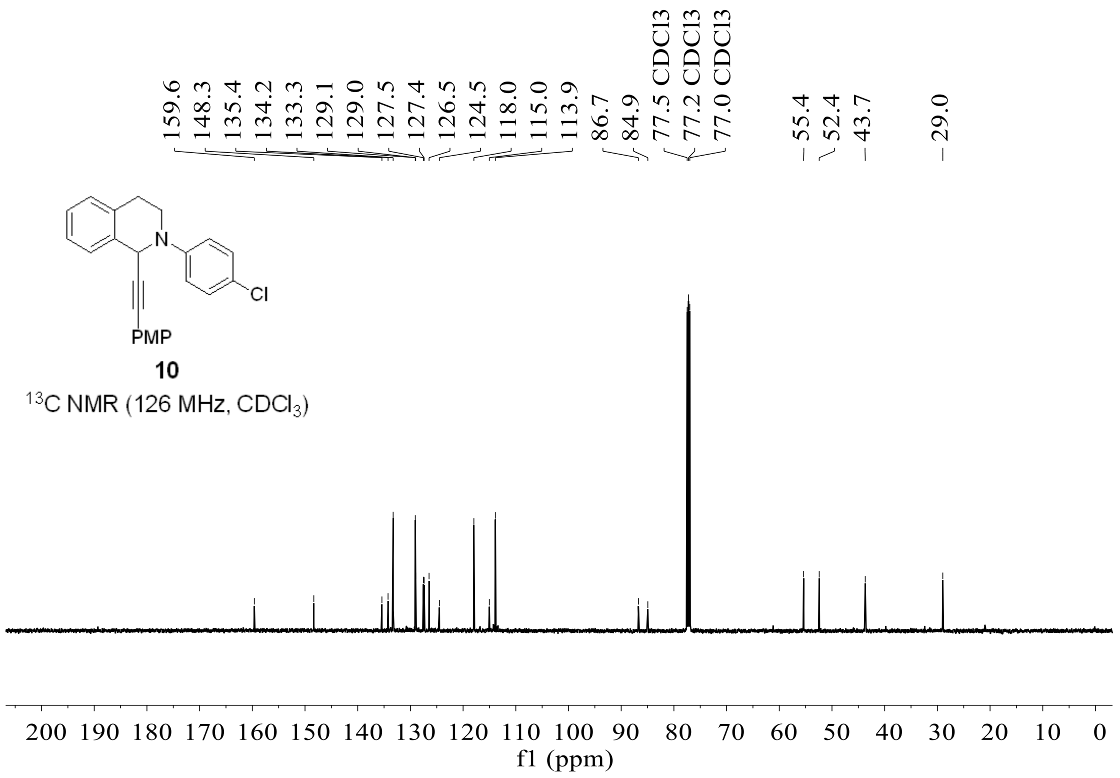

Figure S9. ^{1}H NMR and ^{13}C NMR spectra of compound **10**.

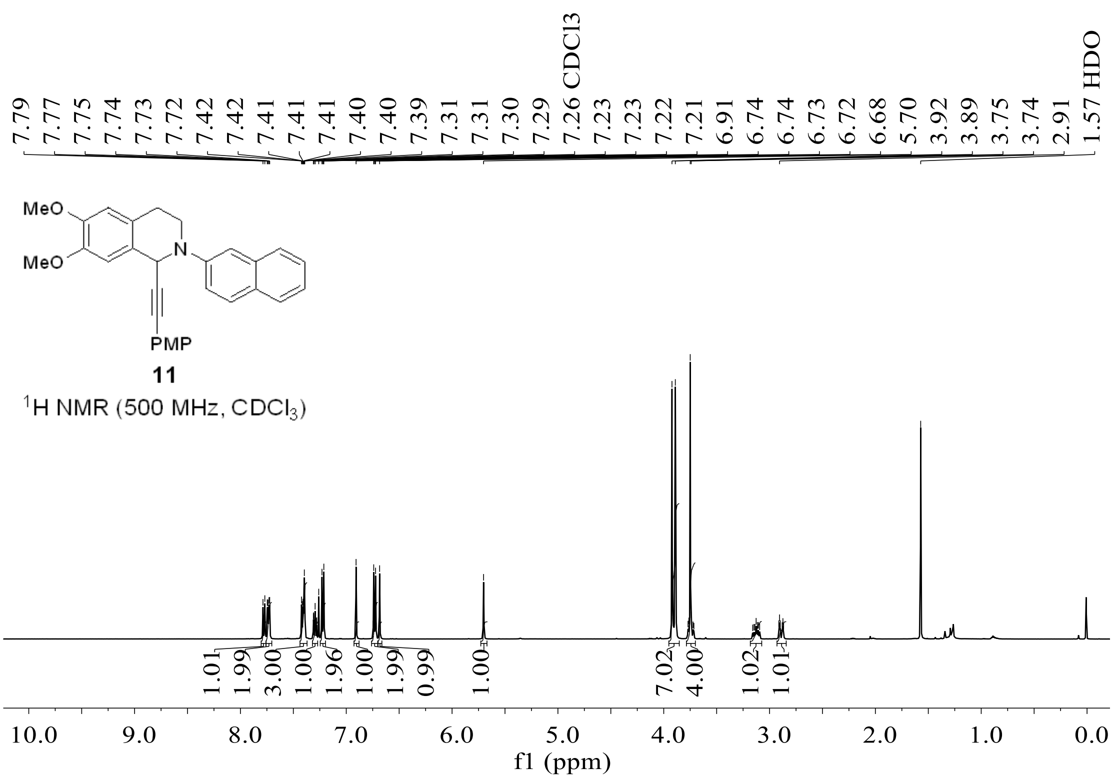

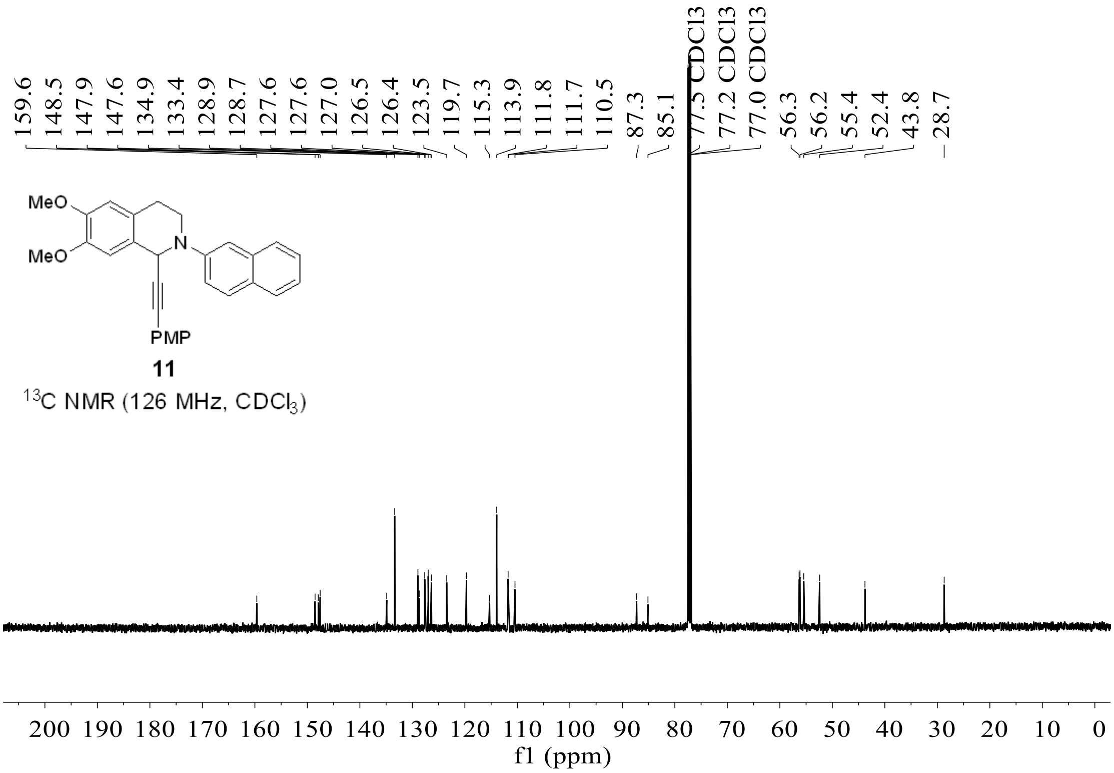

Figure S10. ^{1}H NMR and ^{13}C NMR spectra of compound **11**.

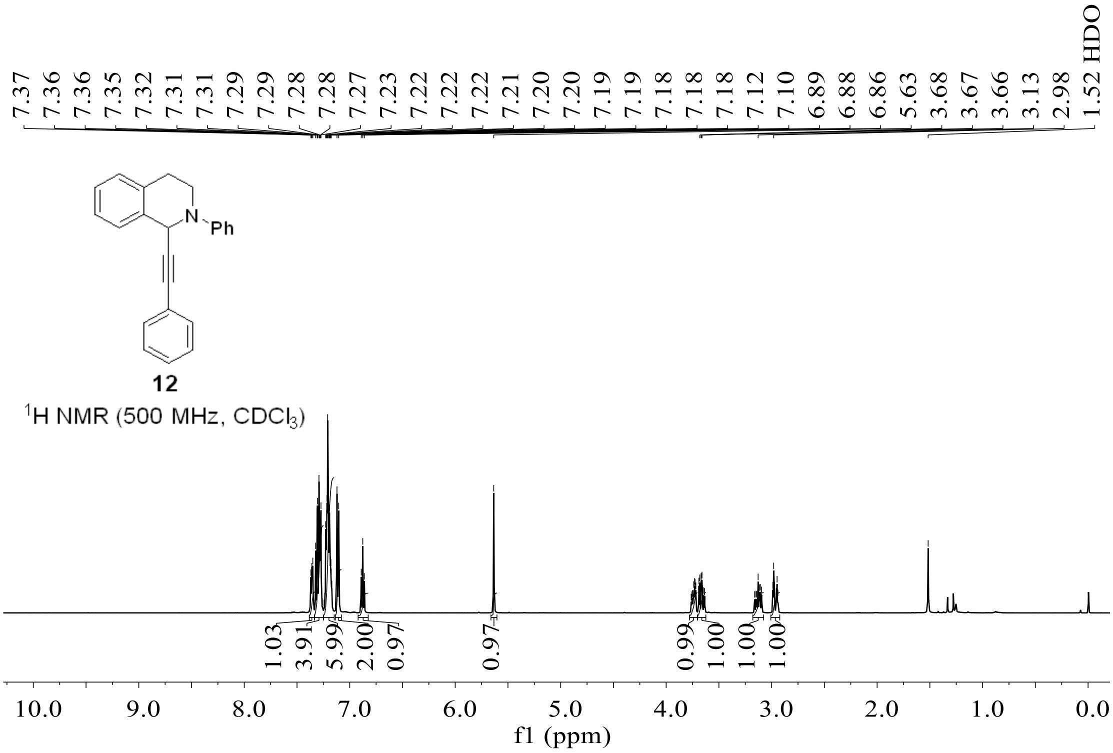

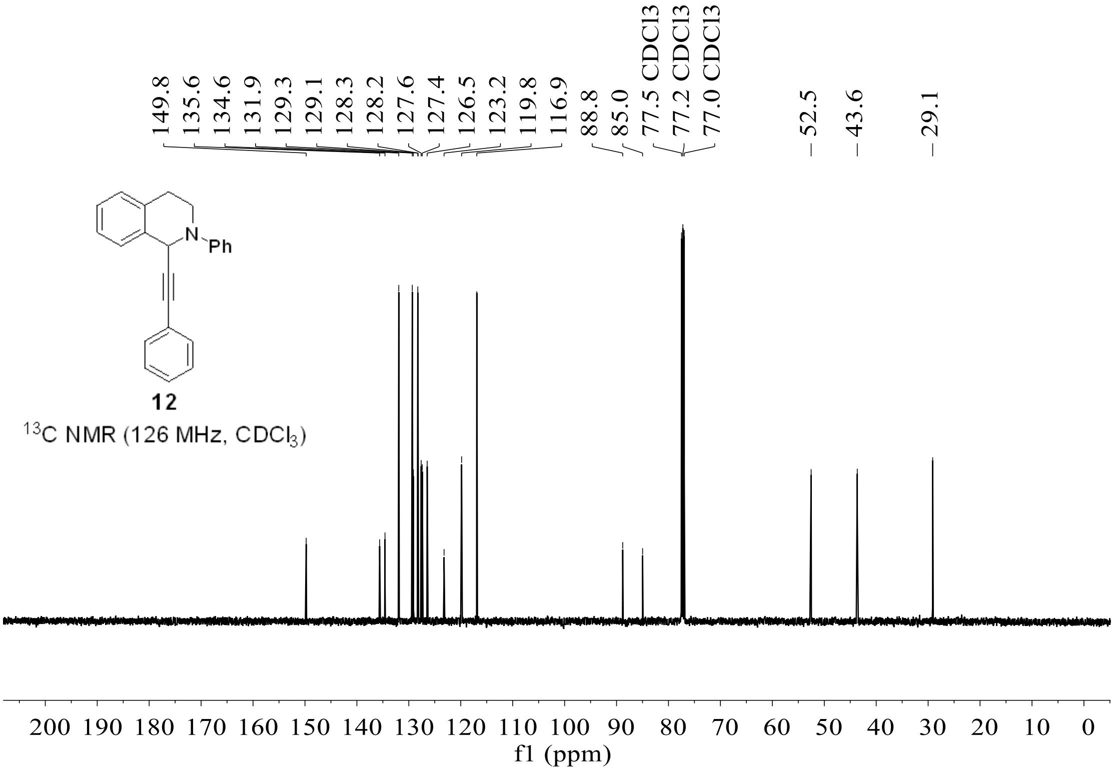

Figure S11. ^{1}H NMR and ^{13}C NMR spectra of compound **12**.

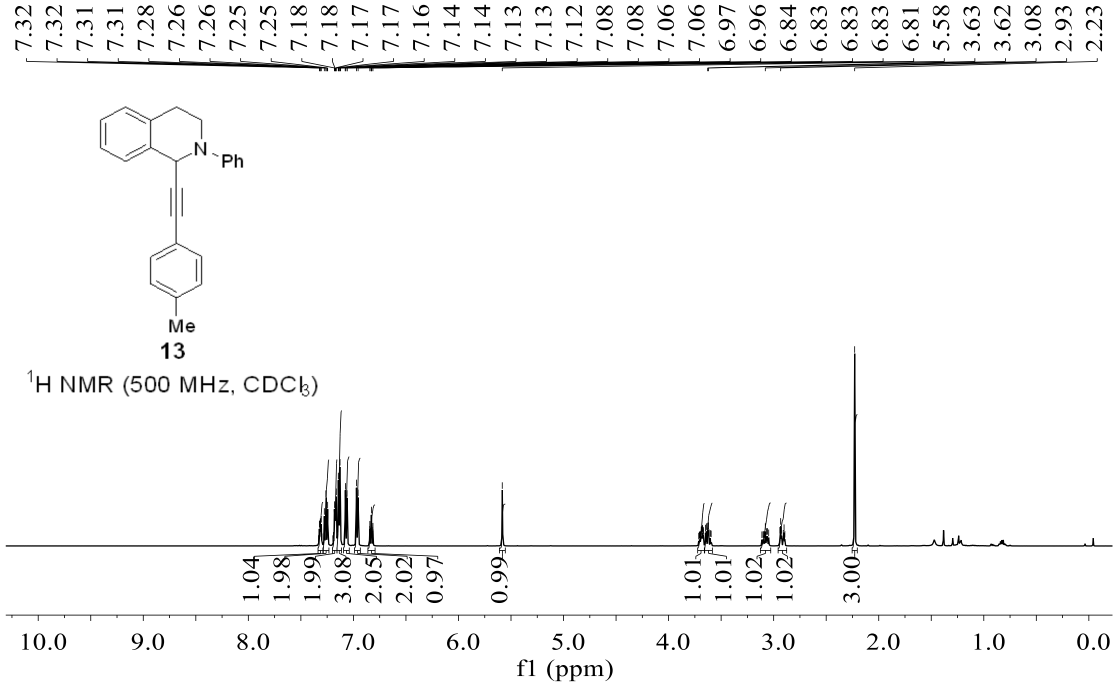

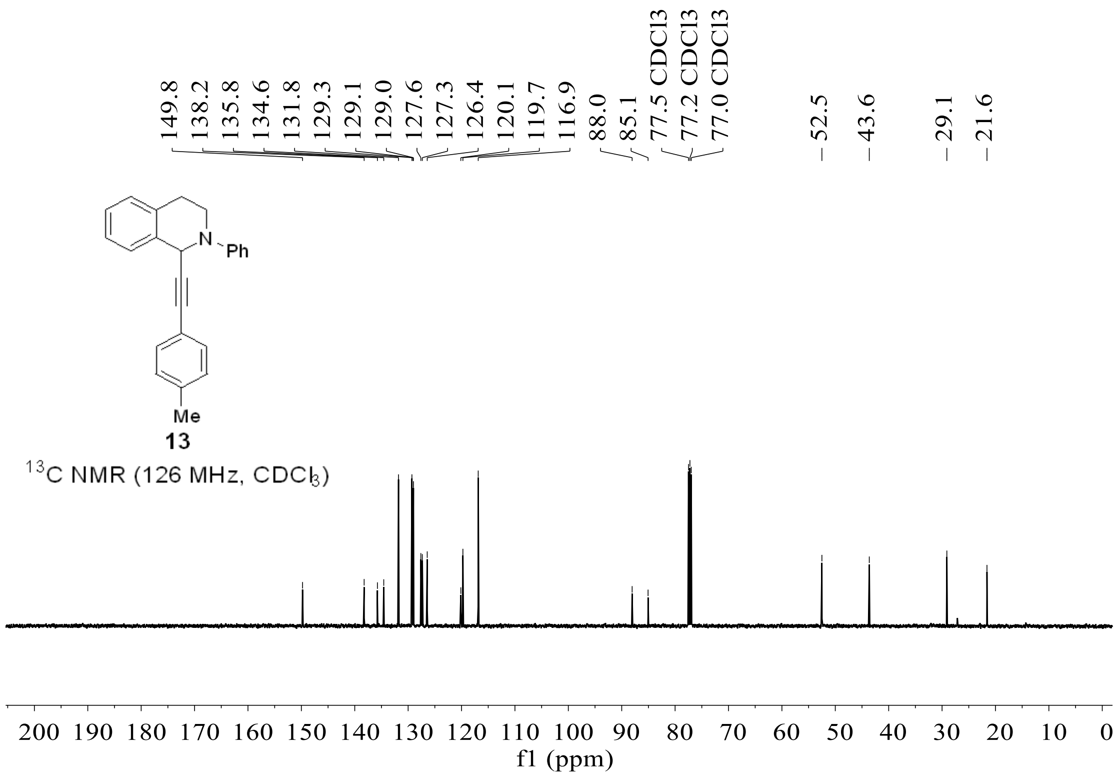

Figure S12. ^{1}H NMR and ^{13}C NMR spectra of compound **13**.

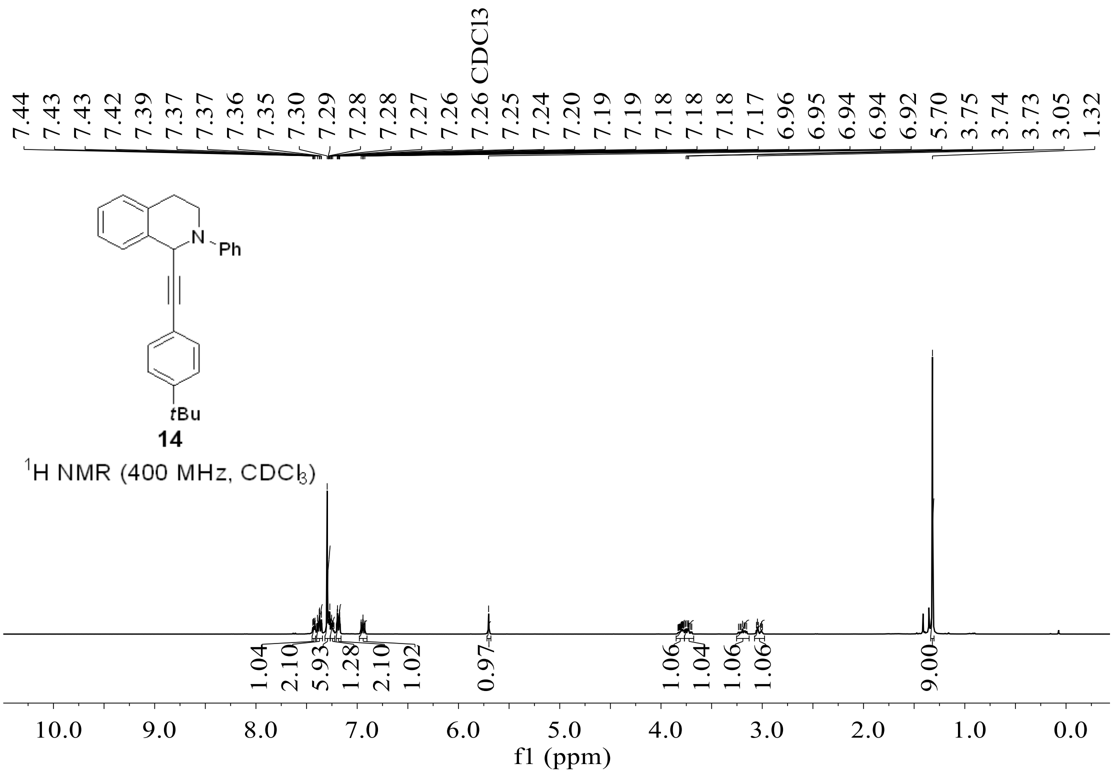

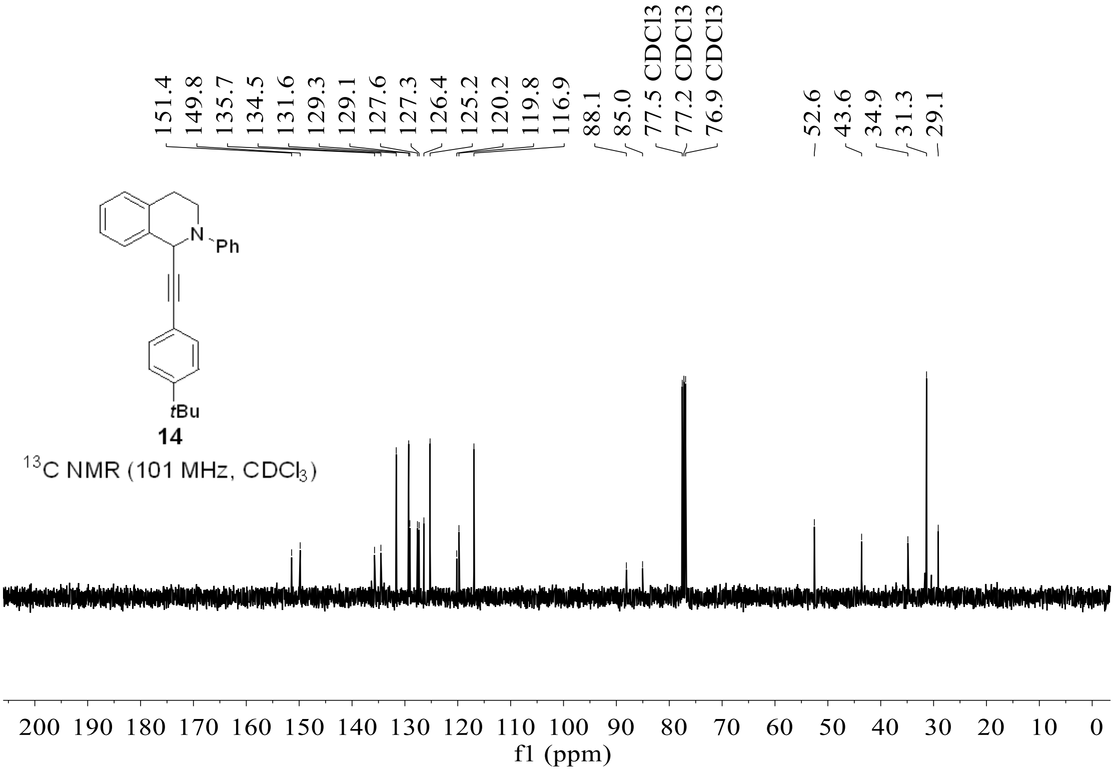

Figure S13. ^{1}H NMR and ^{13}C NMR spectra of compound **14**.

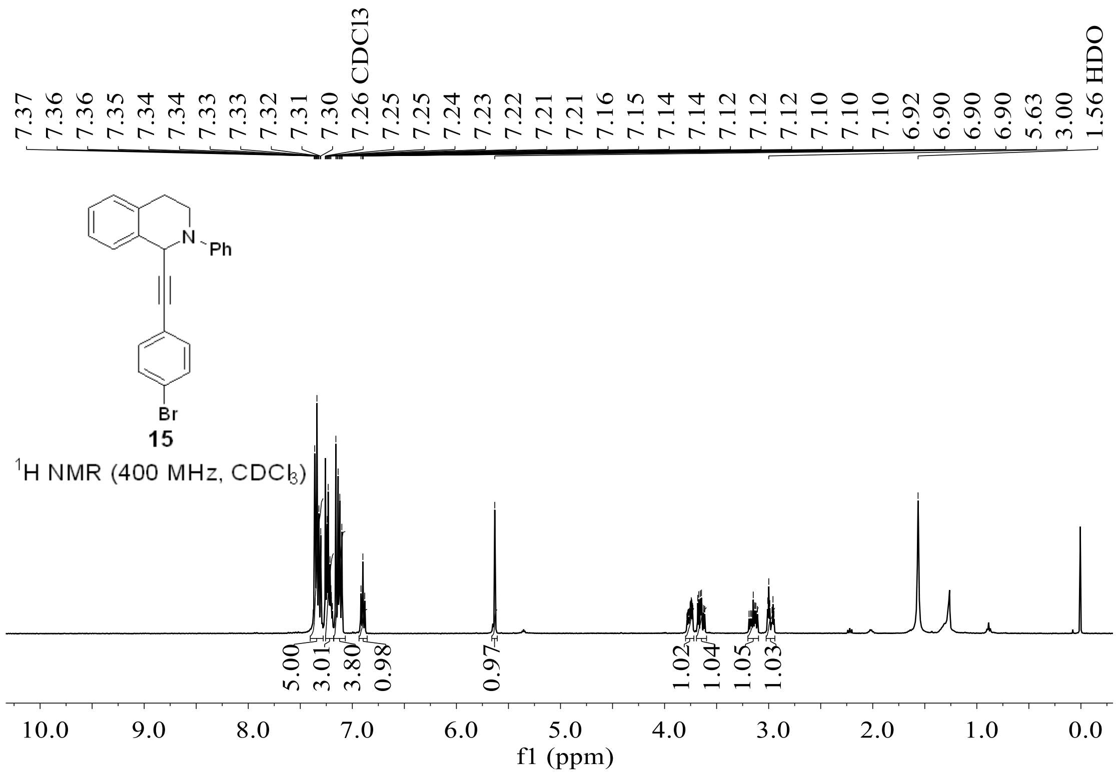

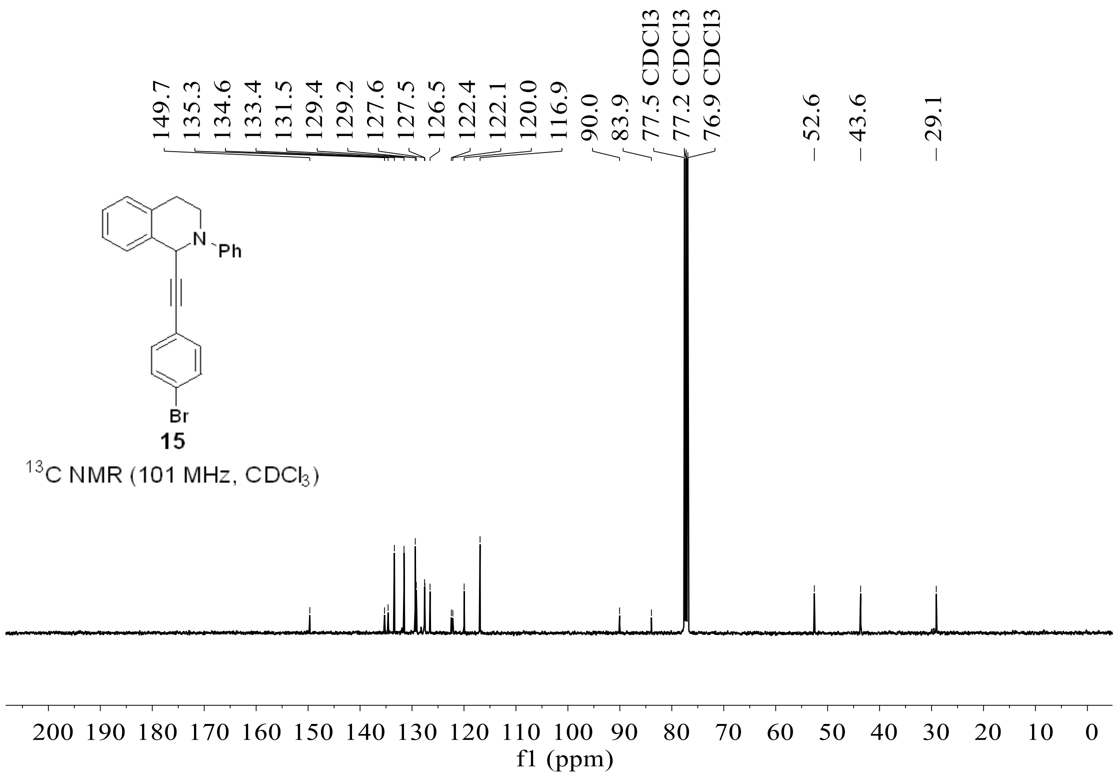

Figure S14. ^{1}H NMR and ^{13}C NMR spectra of compound **15**.

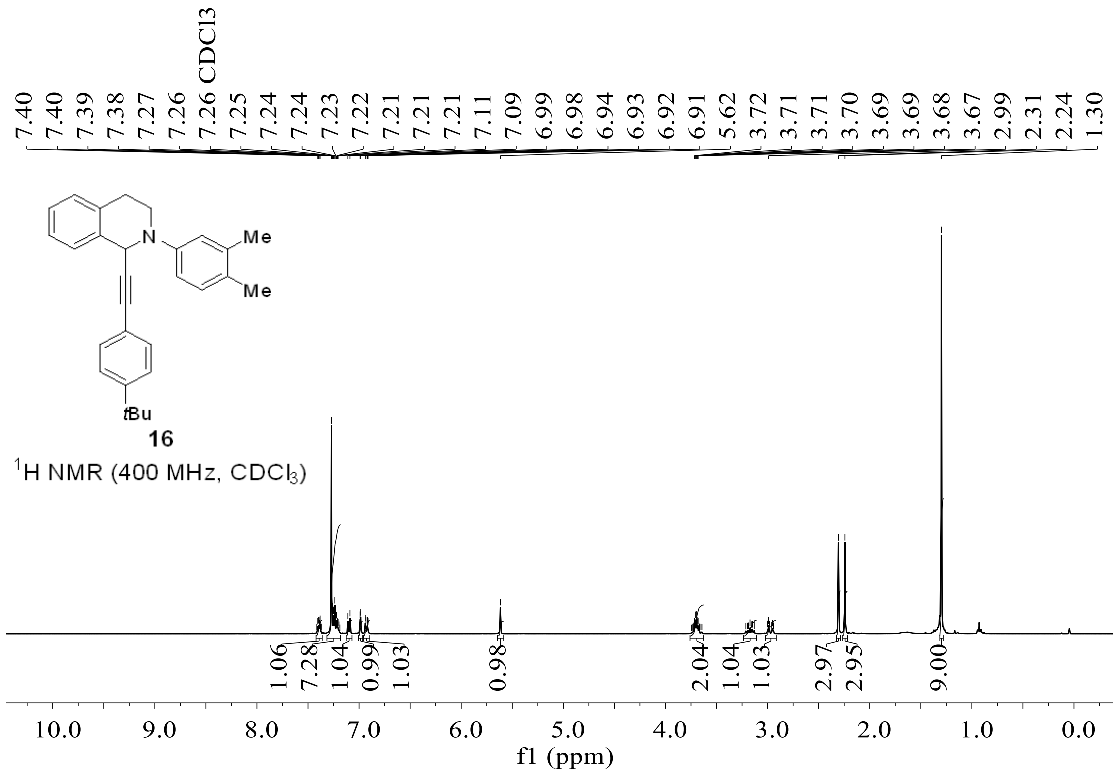

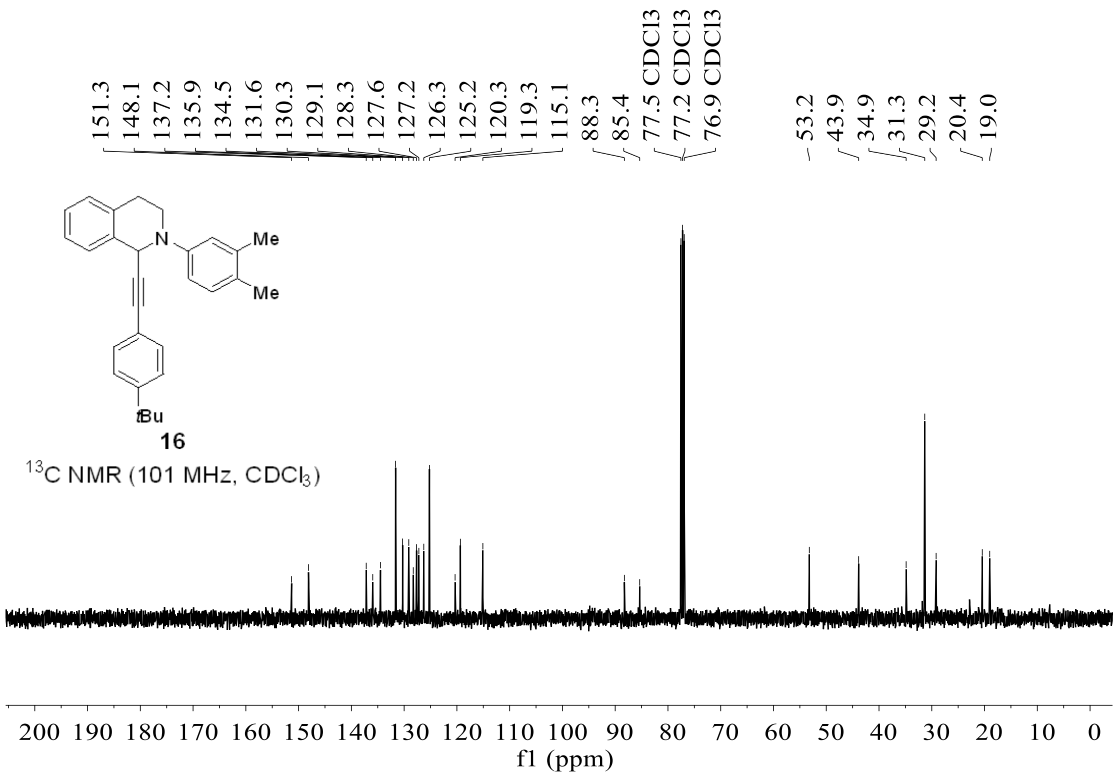

Figure S15. ^{1}H NMR and ^{13}C NMR spectra of compound **16**.

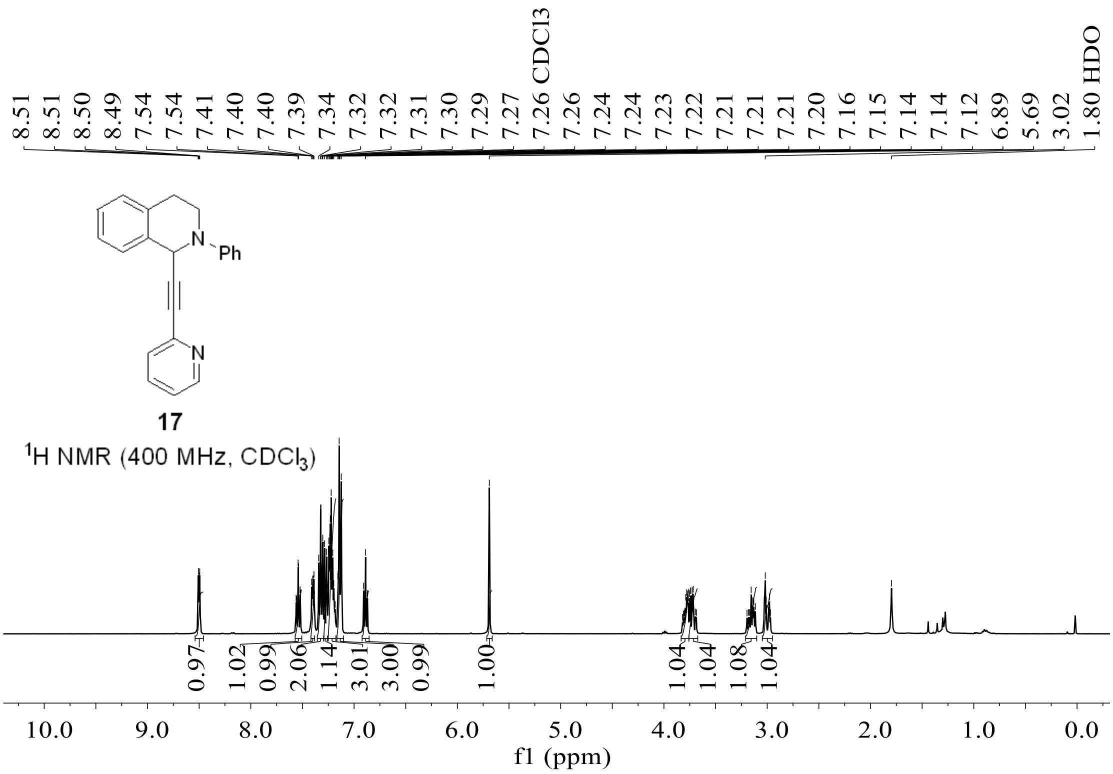

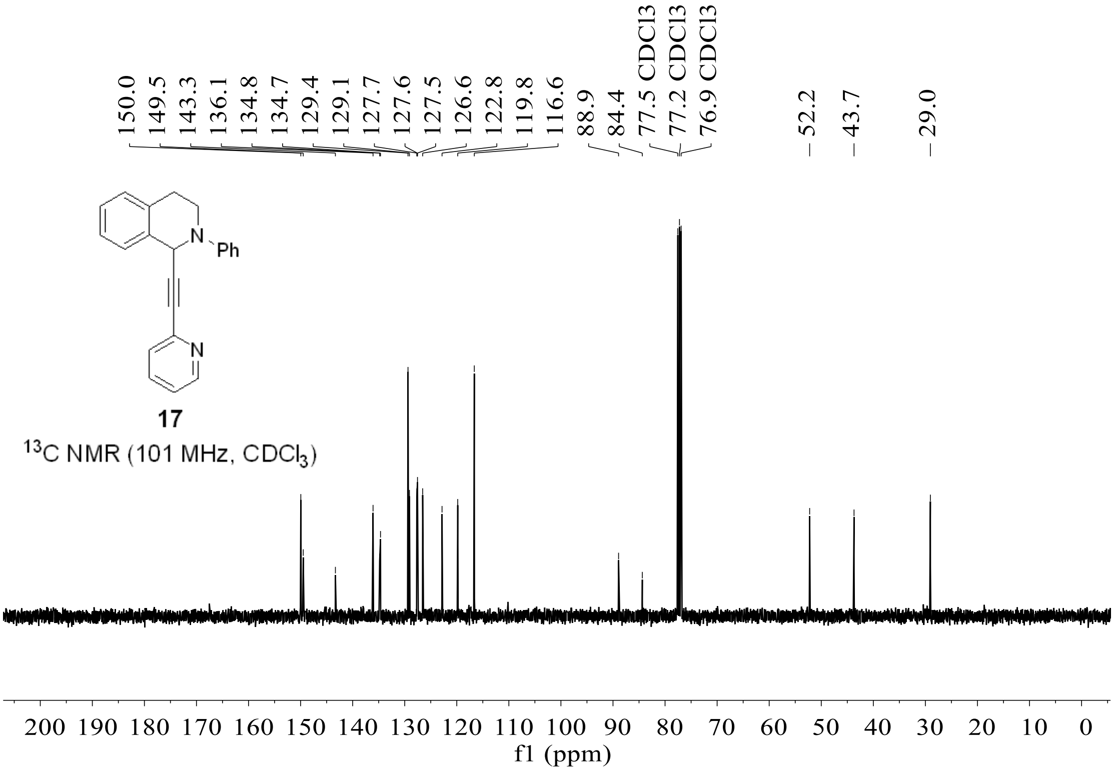

Figure S16. ^{1}H NMR and ^{13}C NMR spectra of compound **17**.

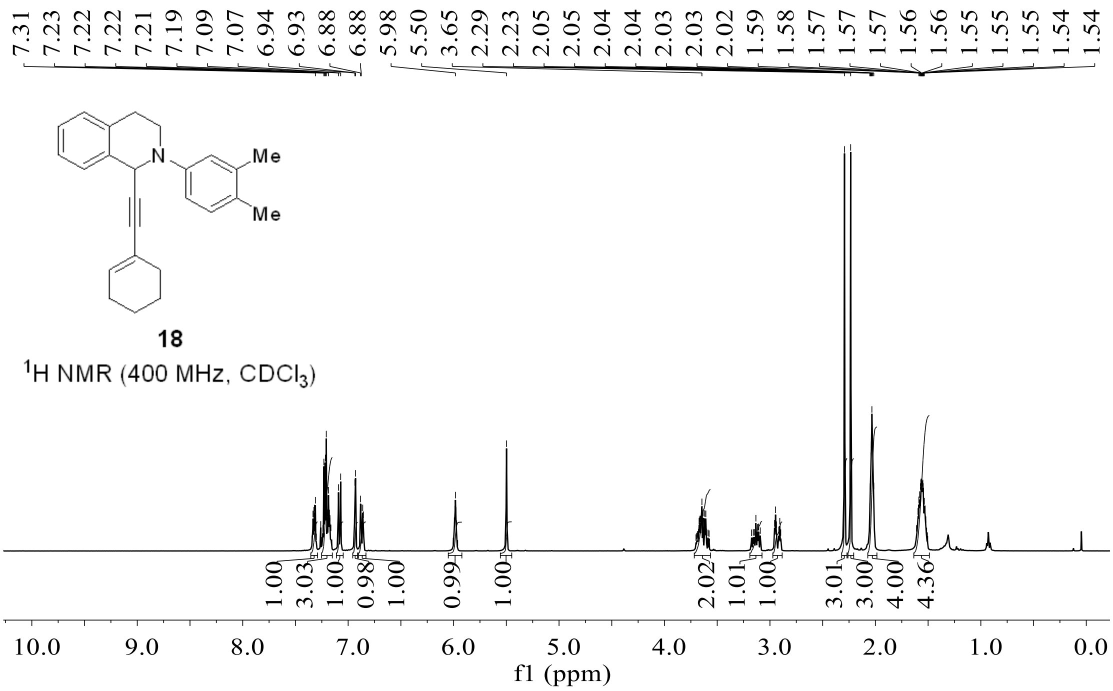

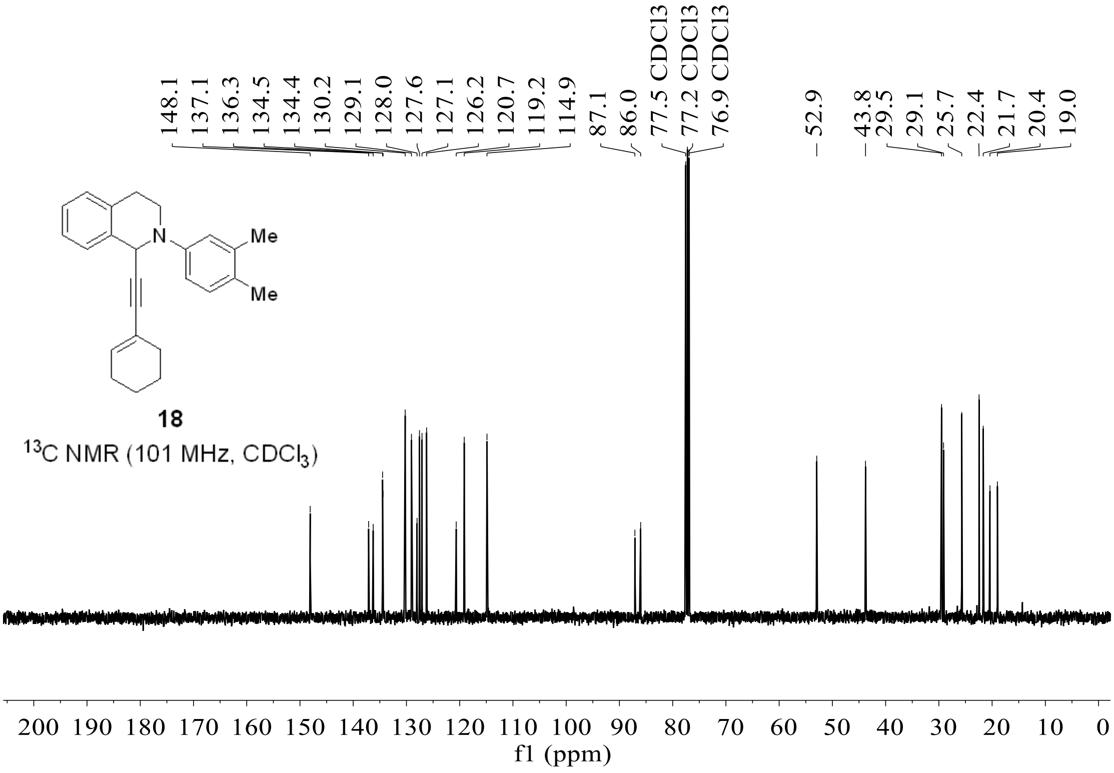

Figure S17. ^{1}H NMR and ^{13}C NMR spectra of compound **18**.

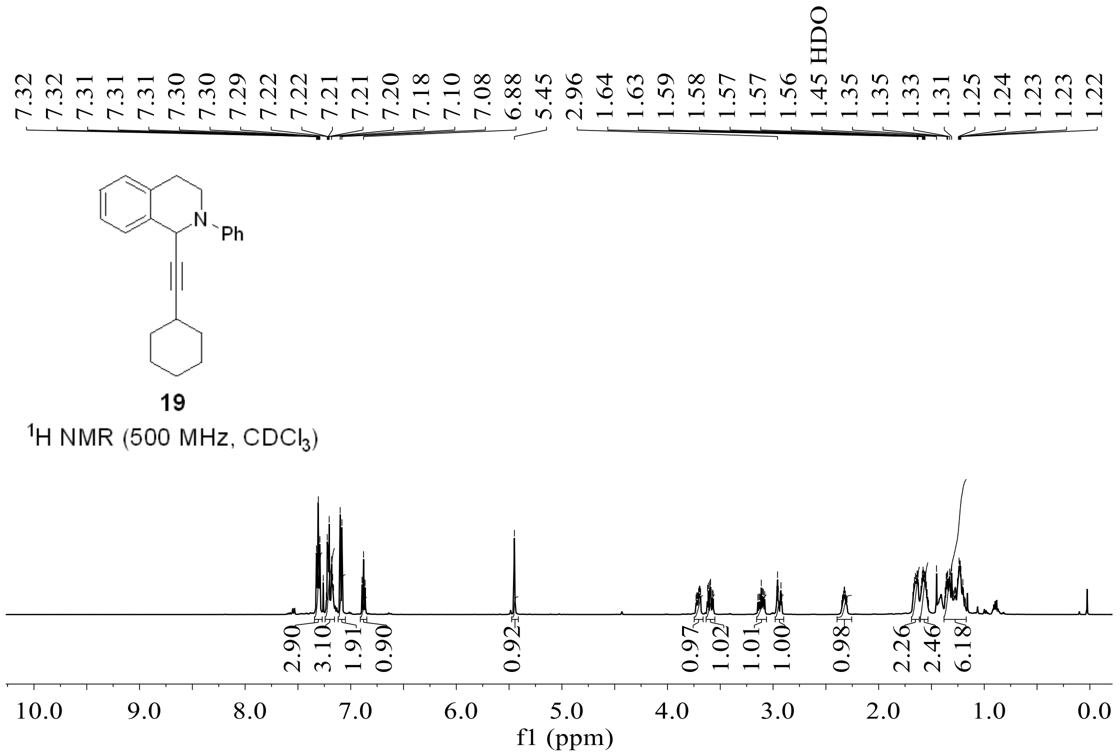

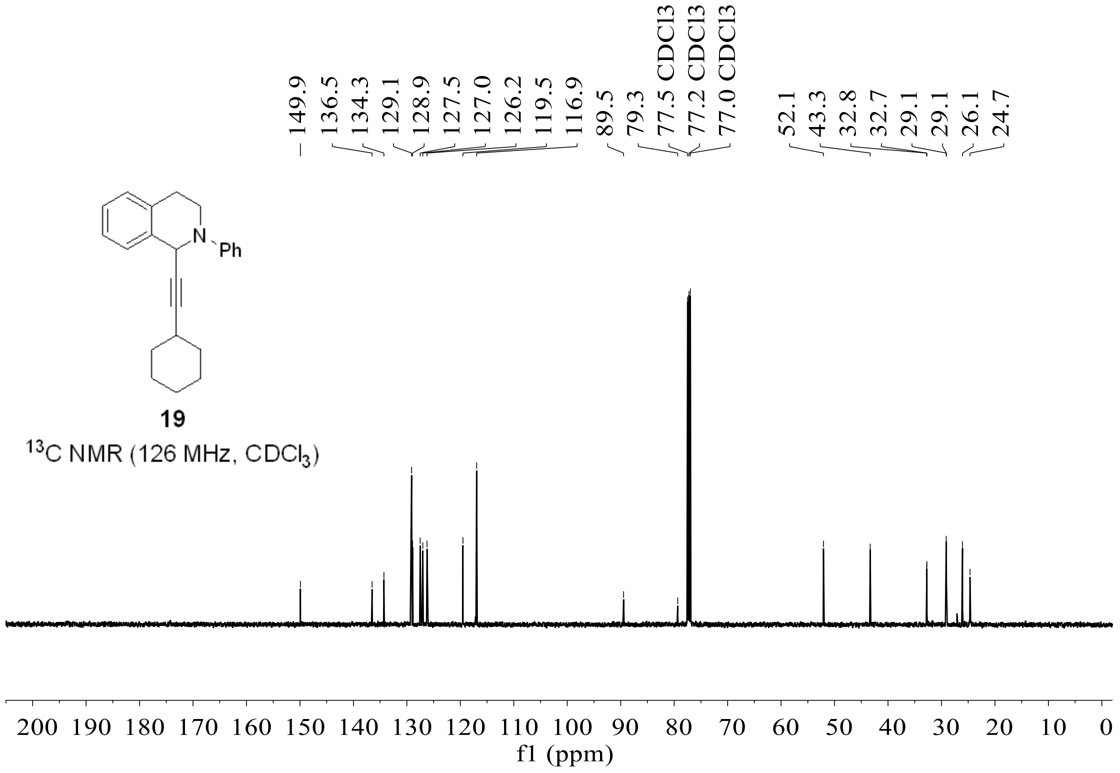

Figure S18. ^{1}H NMR and ^{13}C NMR spectra of compound **19**.

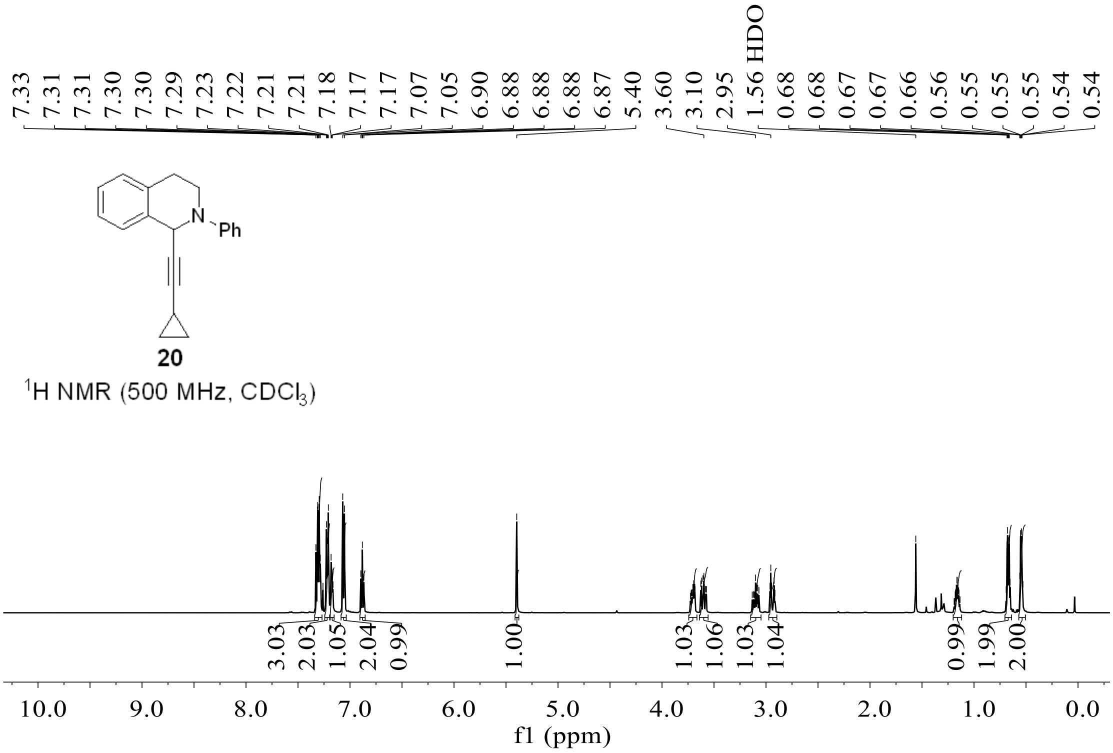

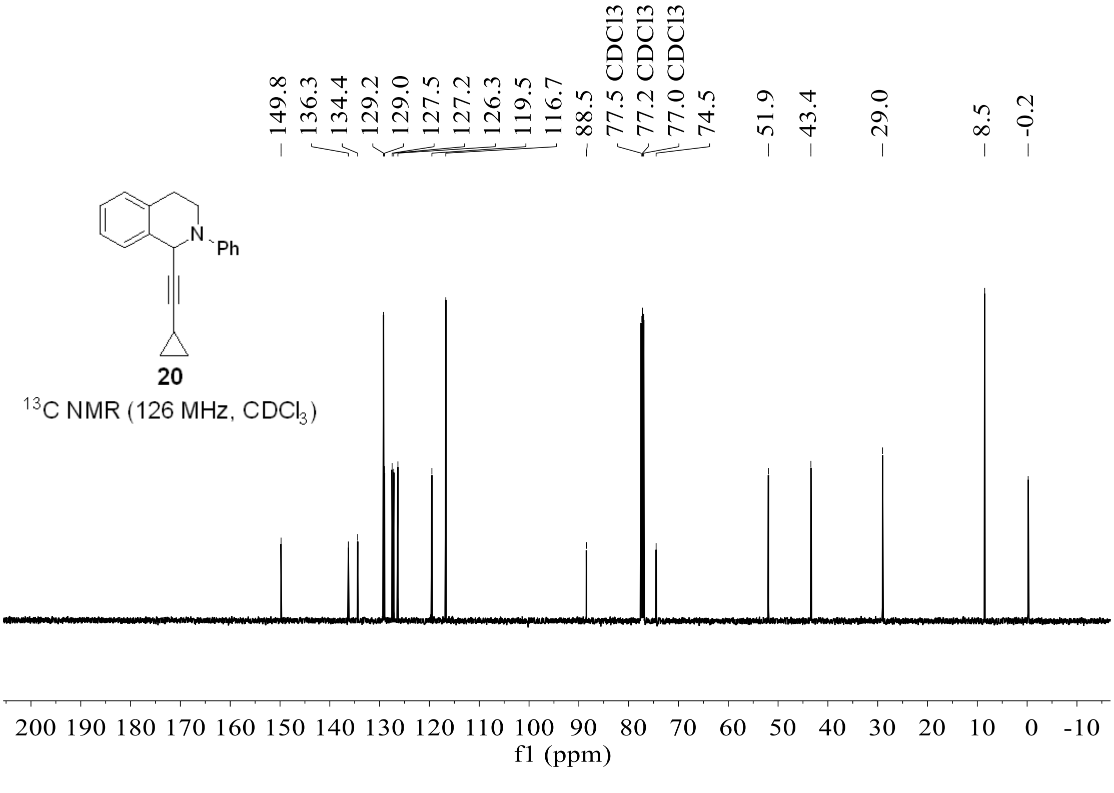

Figure S19. ^{1}H NMR and ^{13}C NMR spectra of compound **20**.

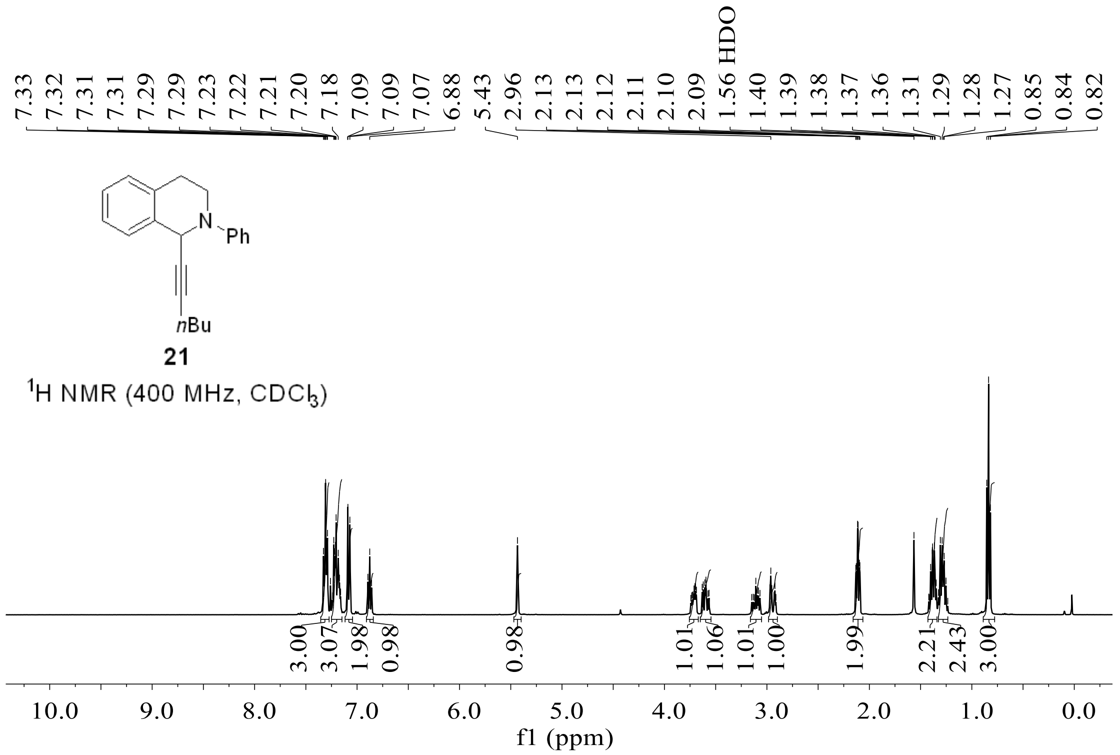

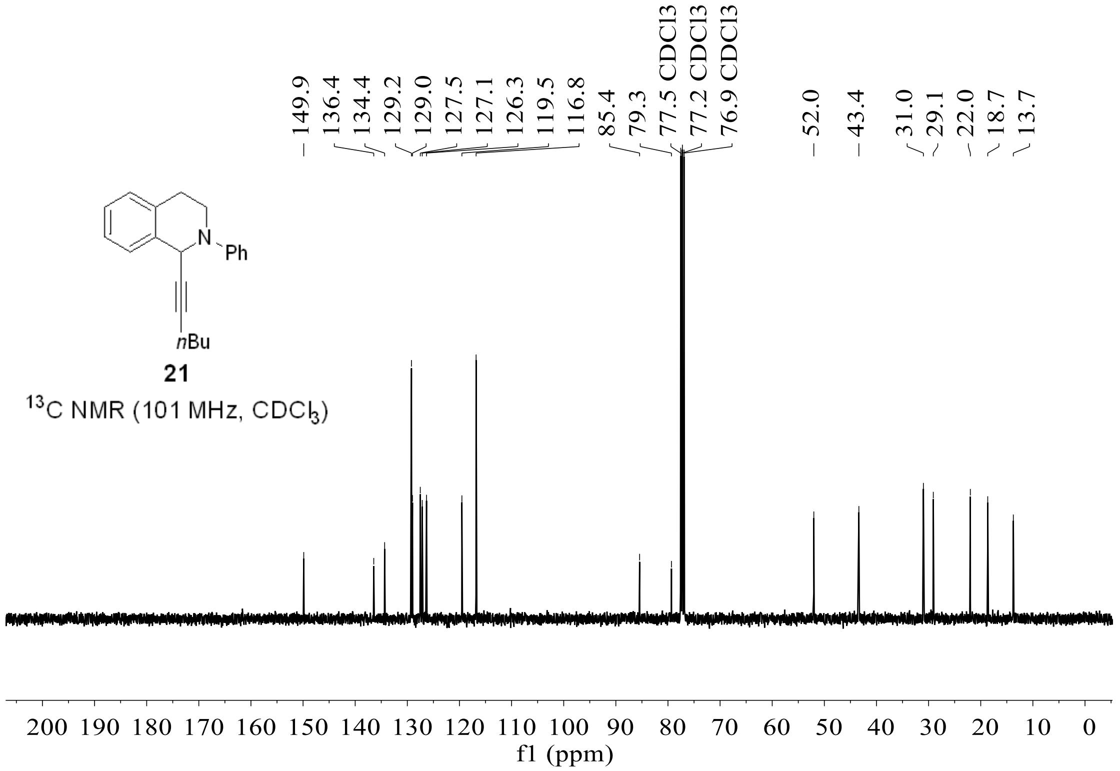

Figure S20. ^{1}H NMR and ^{13}C NMR spectra of compound **21**.

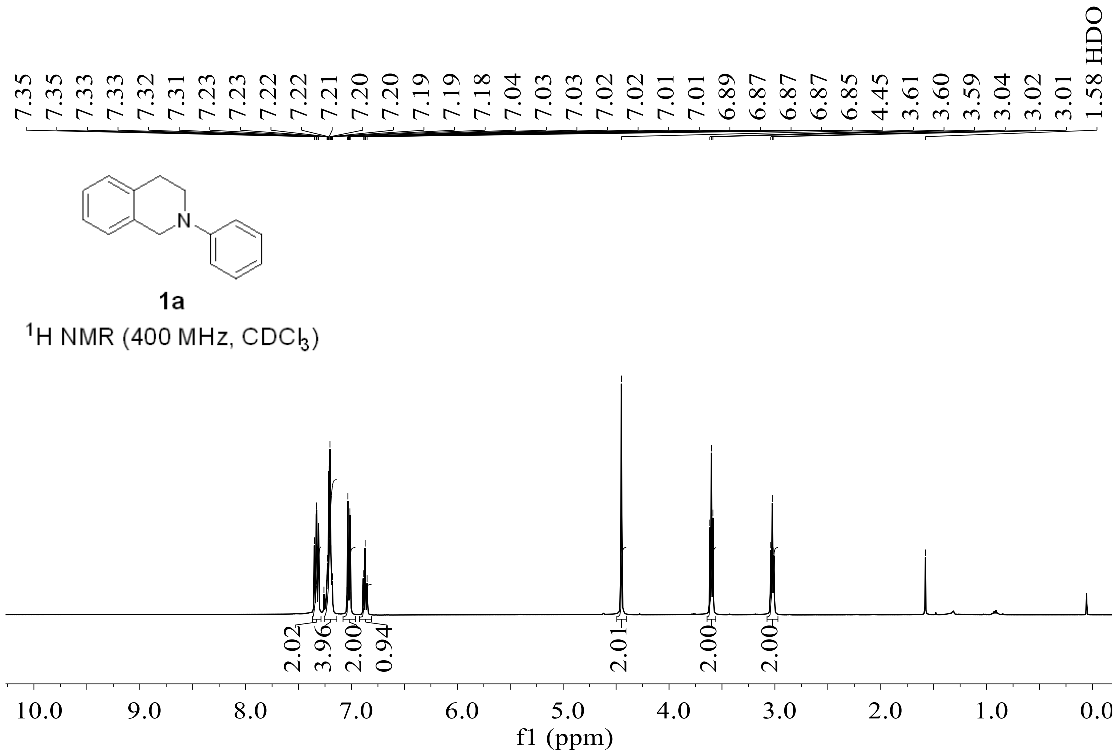

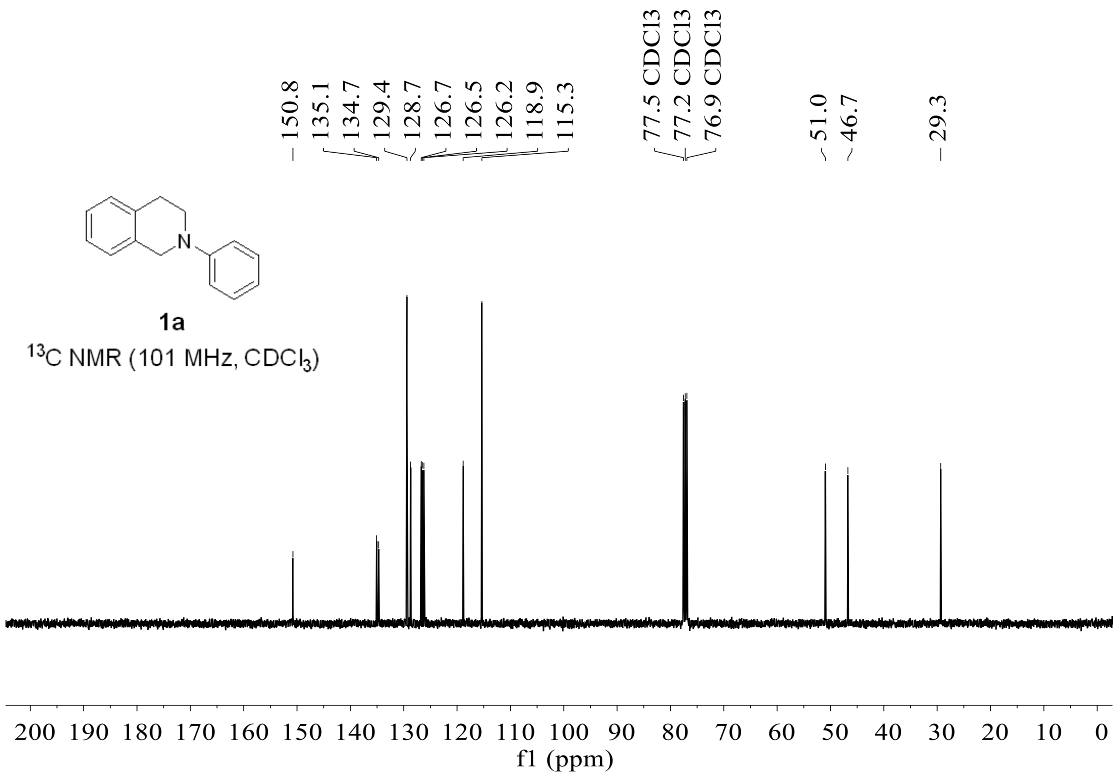

Figure S21. ^{1}H NMR and ^{13}C NMR spectra of compound **1a**.

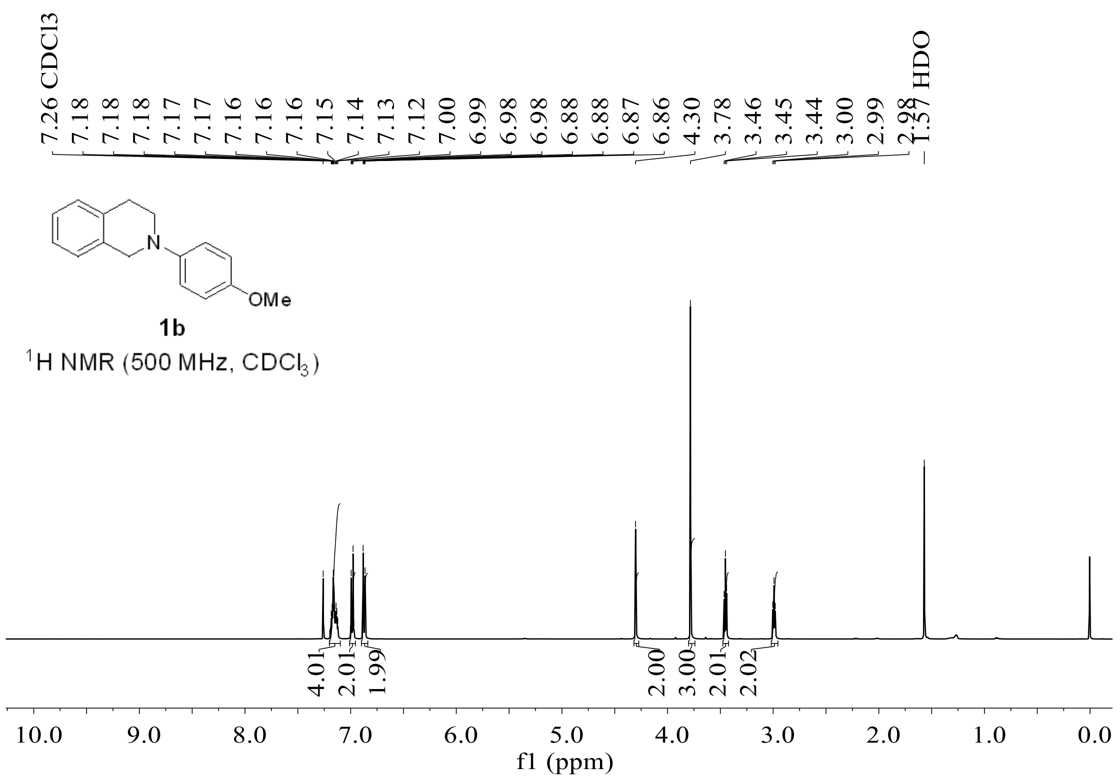

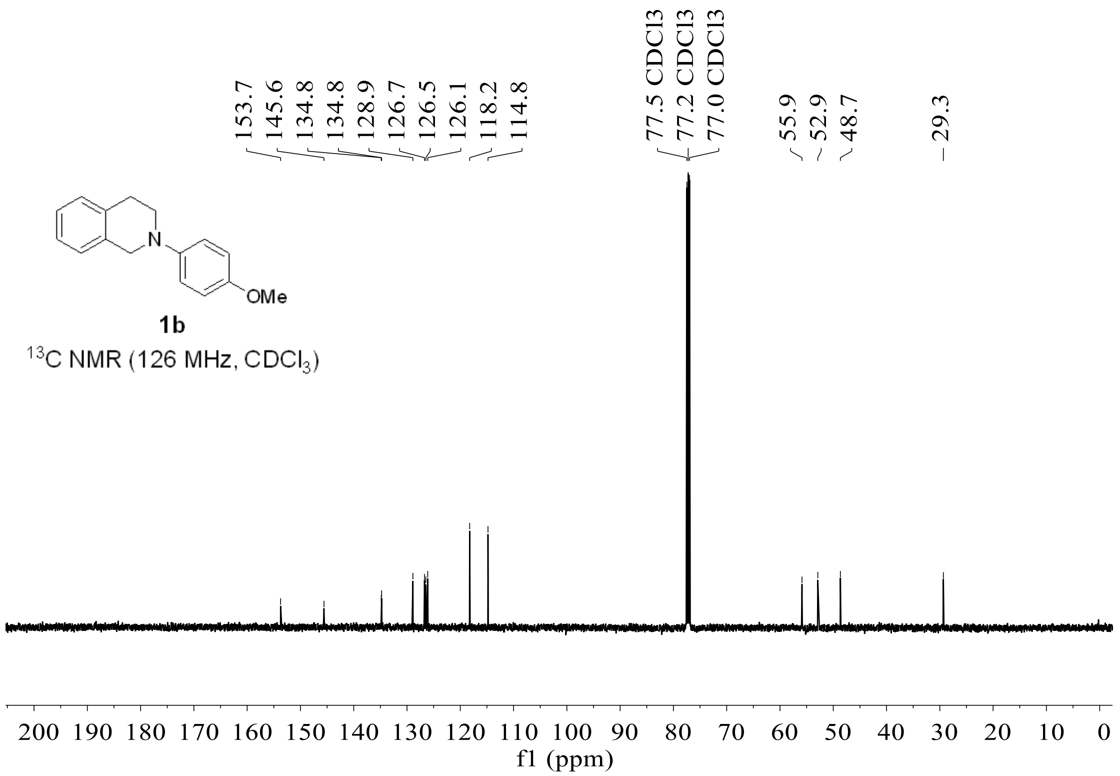

Figure S22. ^{1}H NMR and ^{13}C NMR spectra of compound **1b**.

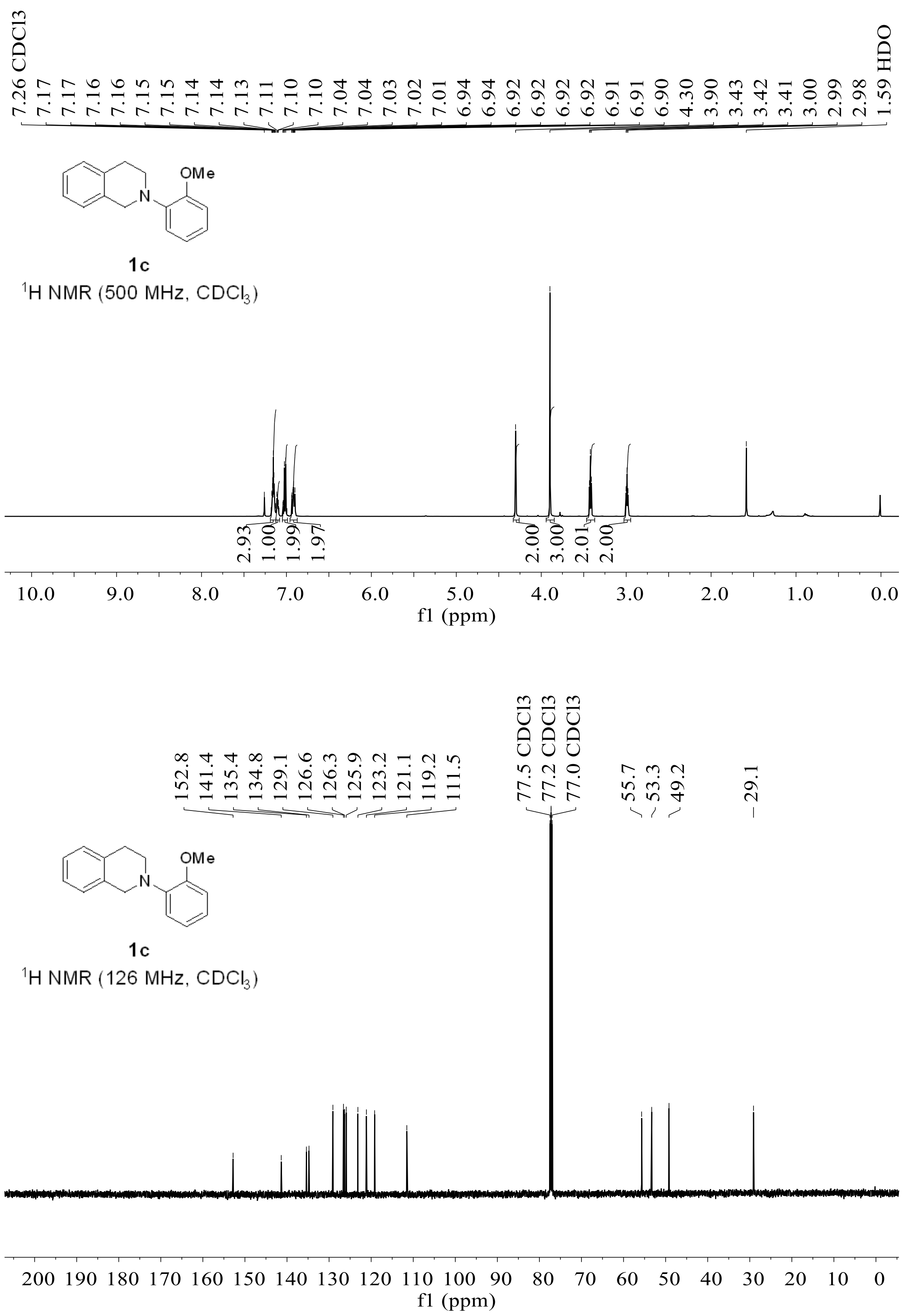

Figure S23. ^{1}H NMR and ^{13}C NMR spectra of compound **1c**.

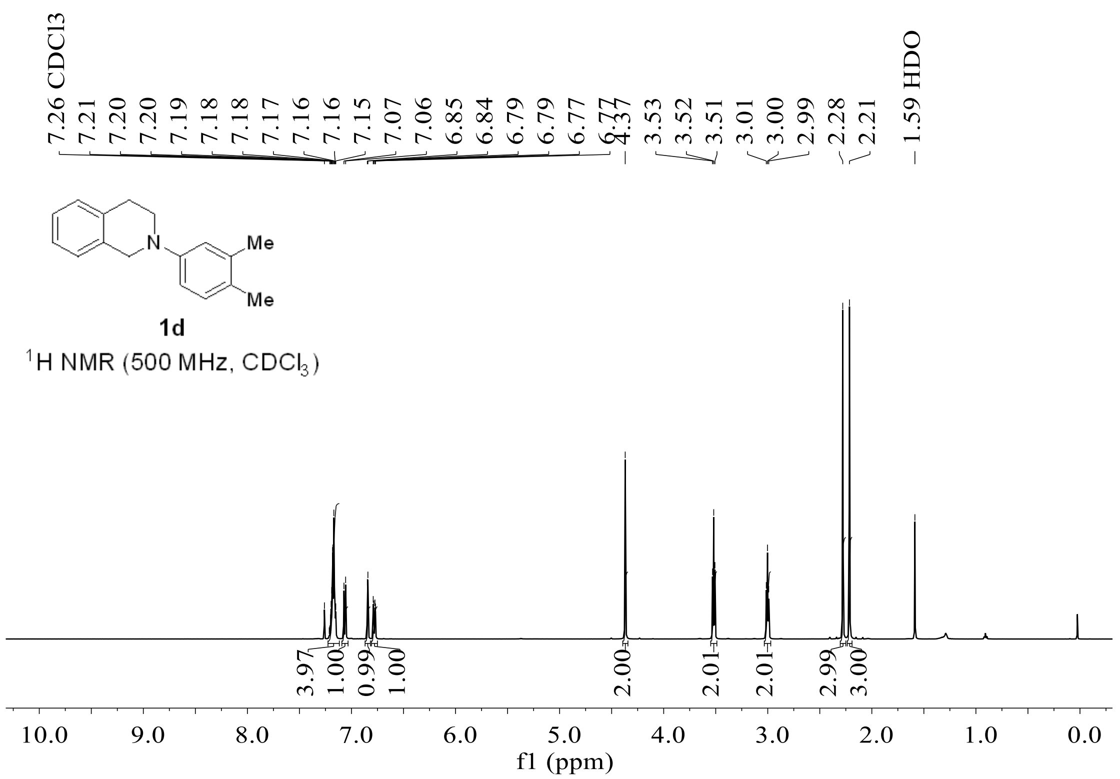

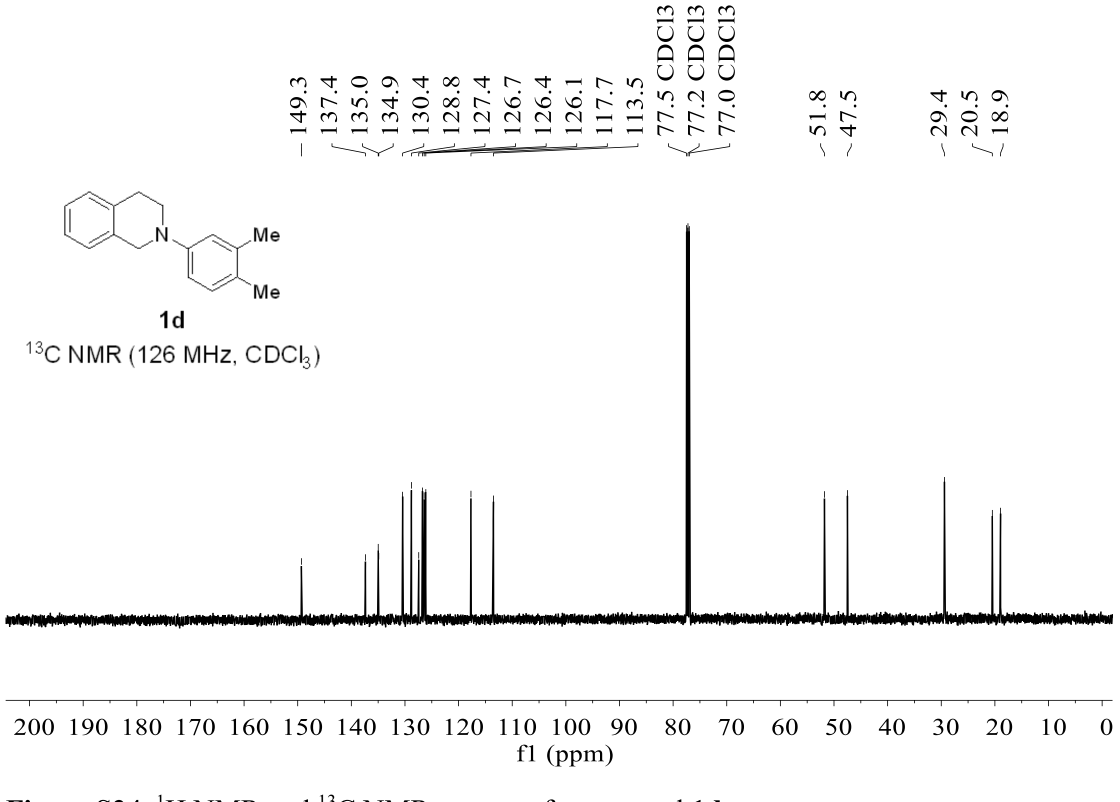

Figure S24. ^{1}H NMR and ^{13}C NMR spectra of compound **1d**.

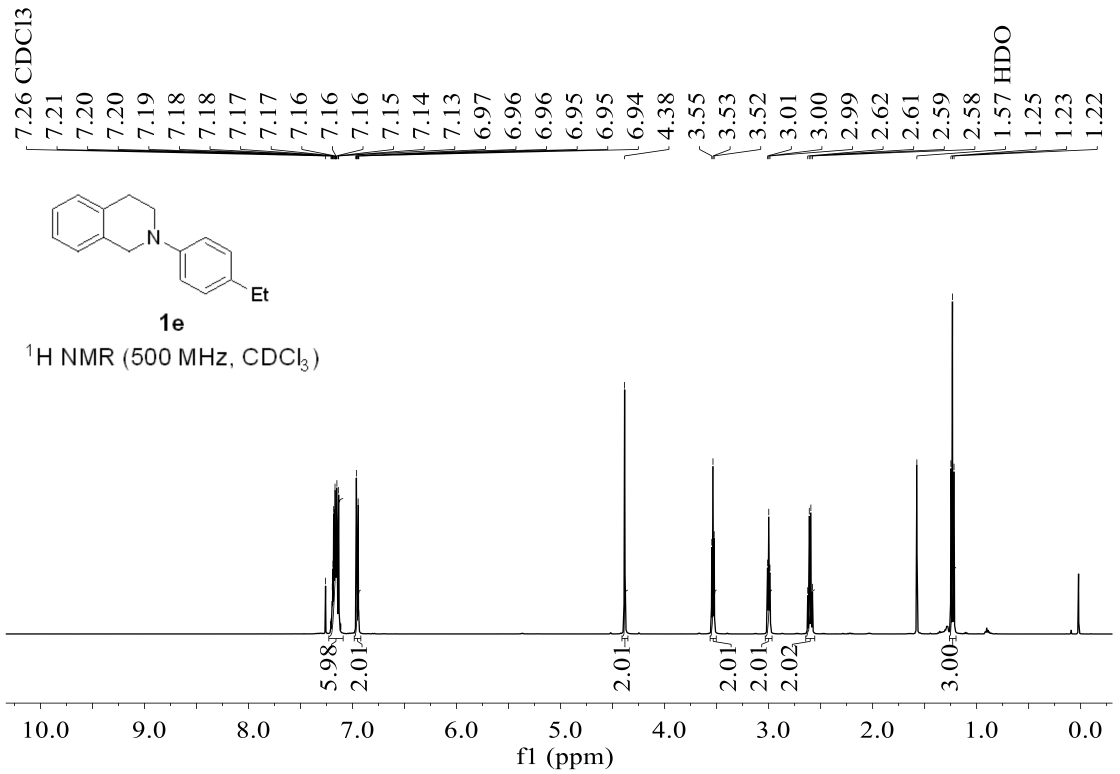

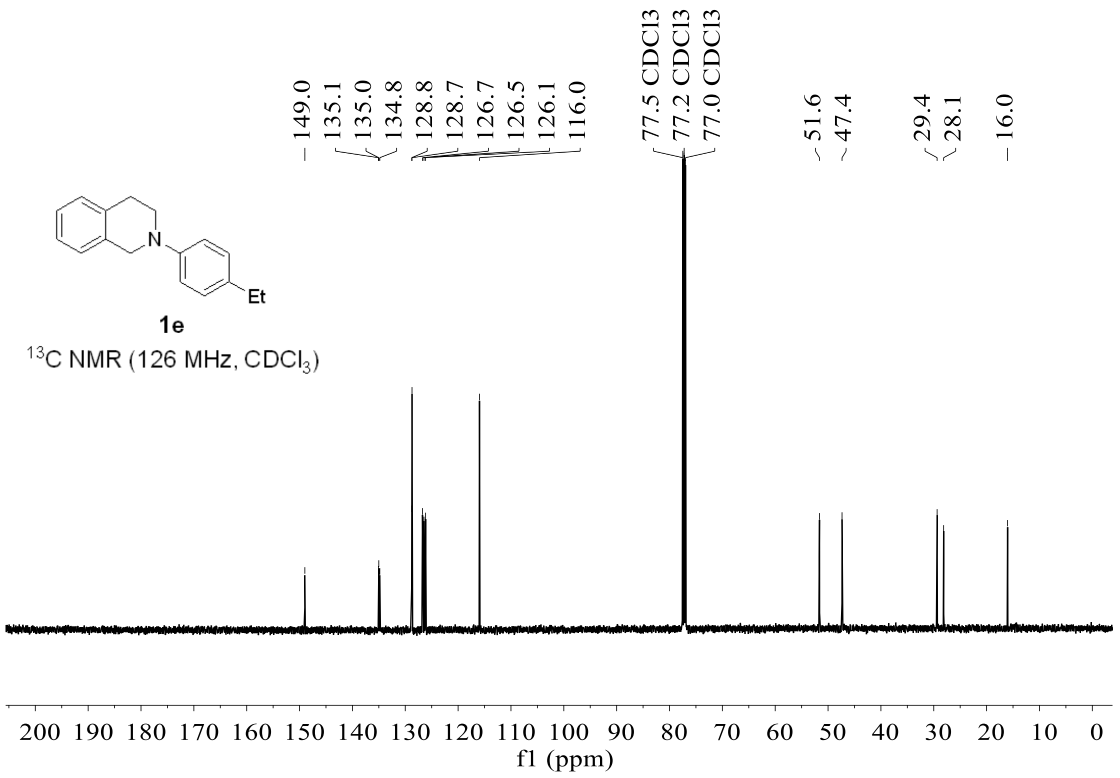

Figure S25. ^{1}H NMR and ^{13}C NMR spectra of compound **1e**.

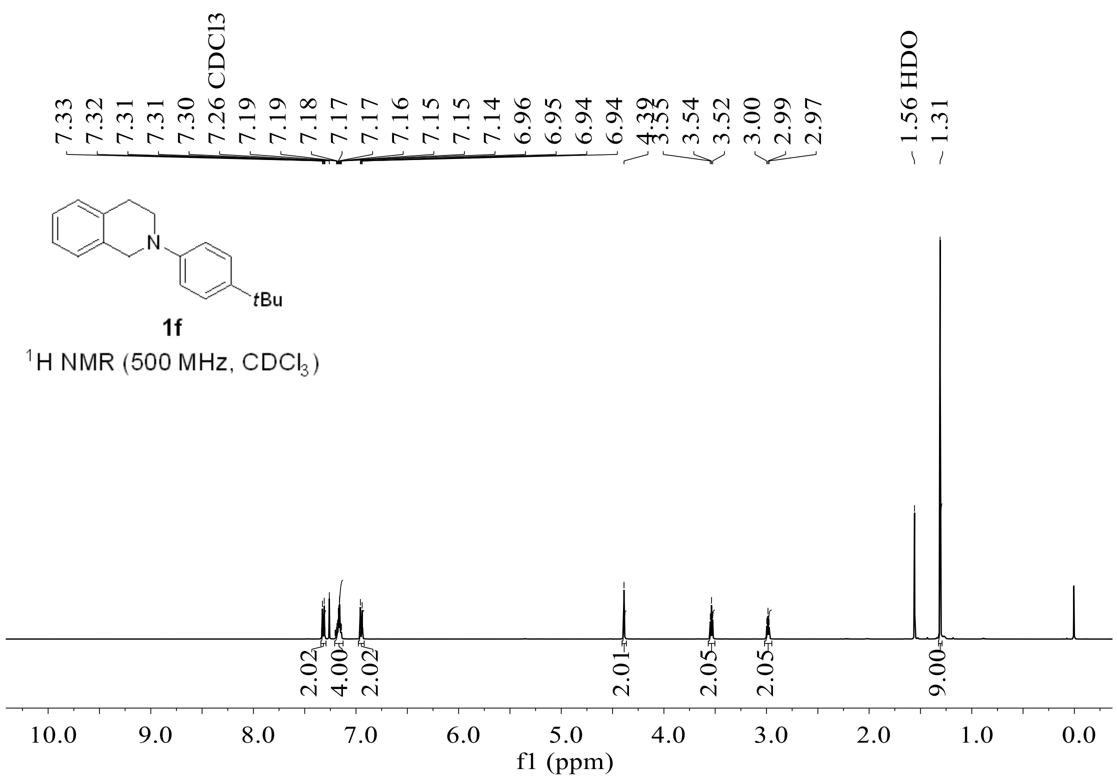

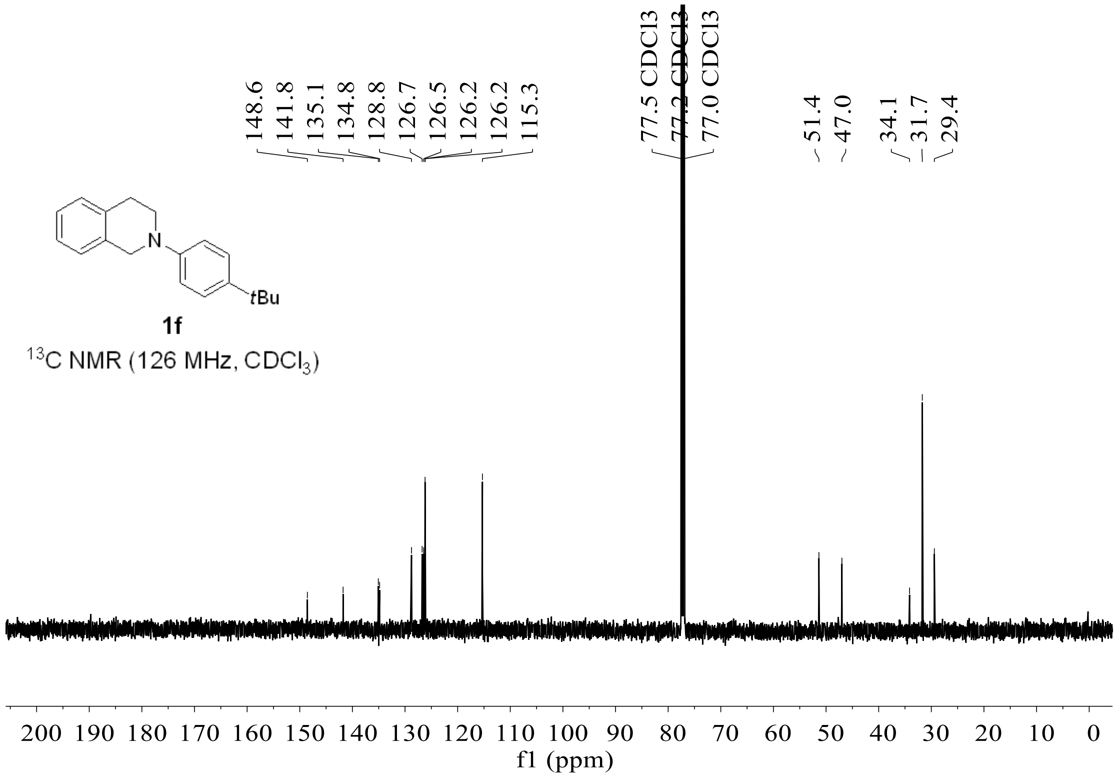

Figure S26. ^{1}H NMR and ^{13}C NMR spectra of compound **1f**.

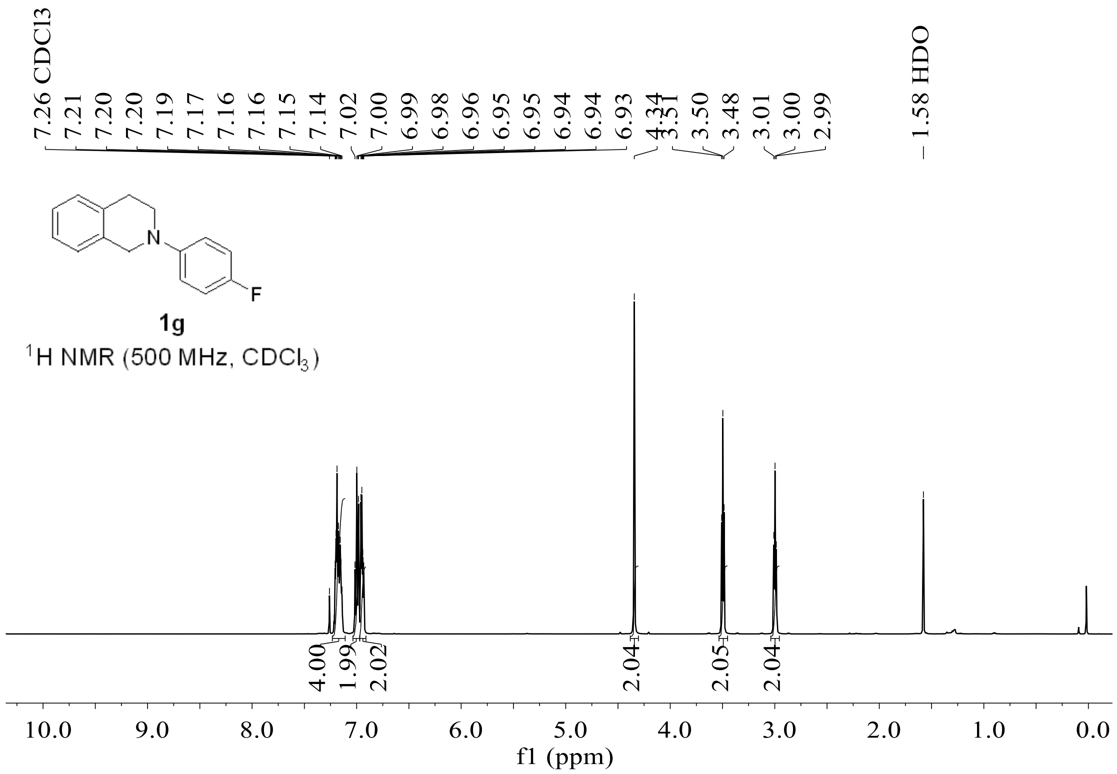
7.26 CDCl3
7.21
7.20
7.20
7.19
7.17
7.16
7.16
7.15
7.14
7.02
7.00
6.99
6.98
6.96
6.95
6.95
6.94
6.94
6.93
4.34
3.51
3.50
3.48
3.01
3.00
2.99
1.58 HDO
N
F
1g
1H NMR (500 MHz, CDCl3)
4.00
1.99
2.02
2.04
2.05
2.04
10.0
9.0
8.0
7.0
6.0
5.0
4.0
3.0
2.0
1.0
0.0
f1 (ppm)

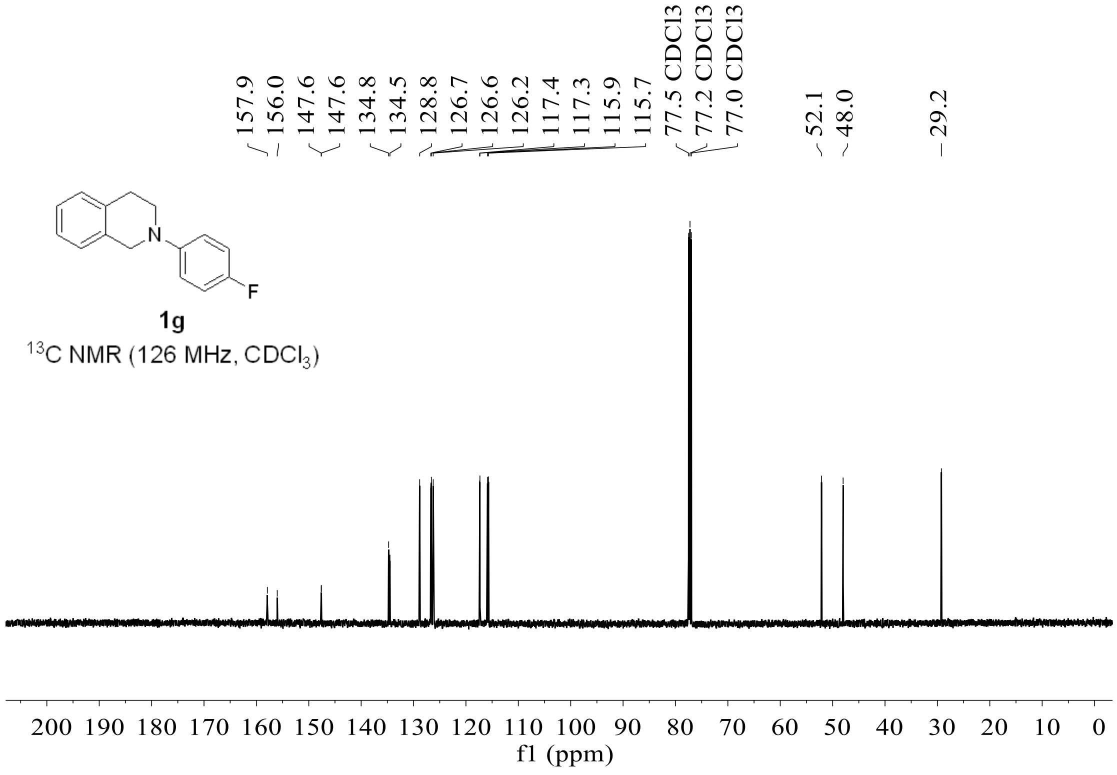
157.9
156.0
147.6
147.6
134.8
134.5
128.8
126.7
126.6
126.2
117.4
117.3
115.9
115.7
77.5 CDCl3
77.2 CDCl3
77.0 CDCl3
52.1
48.0
29.2
N
F
1g
13C NMR (126 MHz, CDCl3)
200
190
180
170
160
150
140
130
120
110
100
90
80
70
60
50
40
30
20
10
0
f1 (ppm)

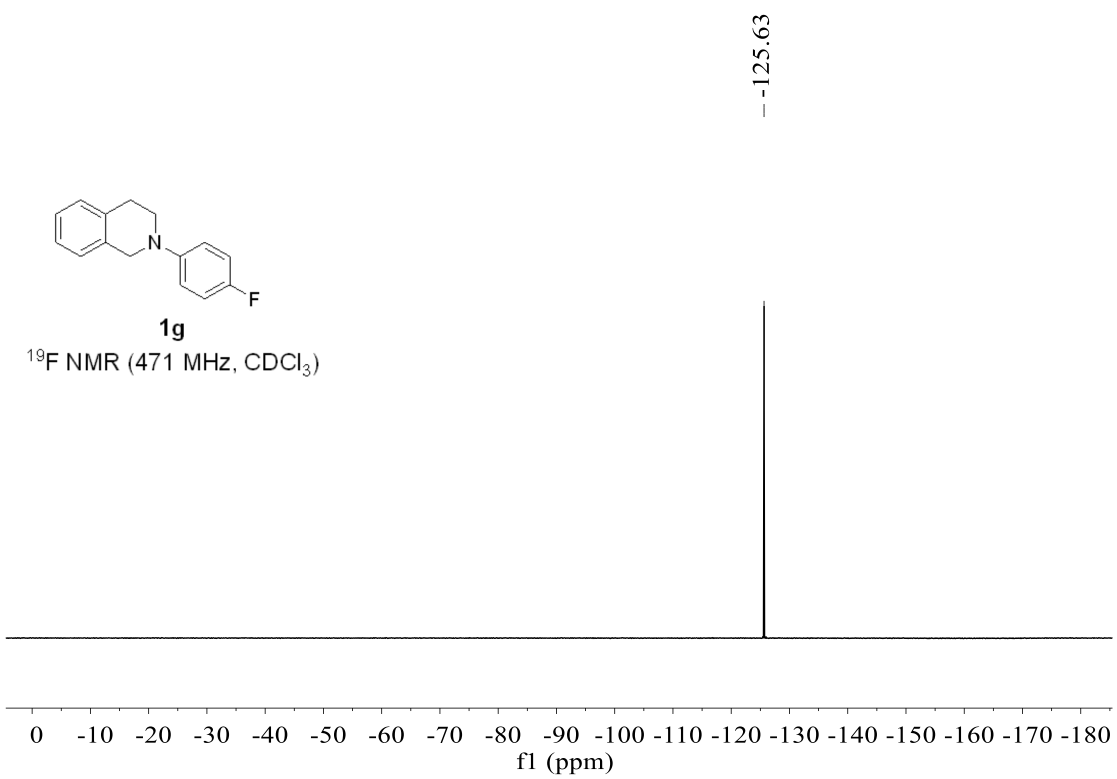

Figure S27. ^{1}H NMR, ^{13}C NMR and ^{19}F NMR spectra of compound **1g**.

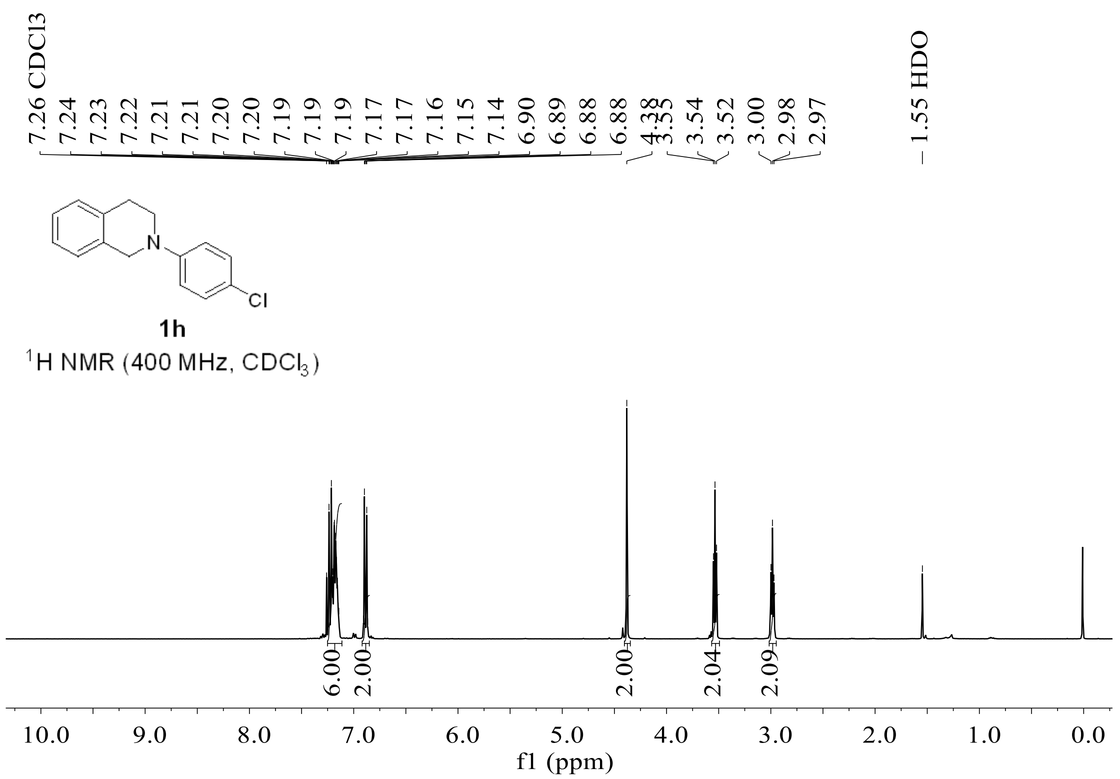

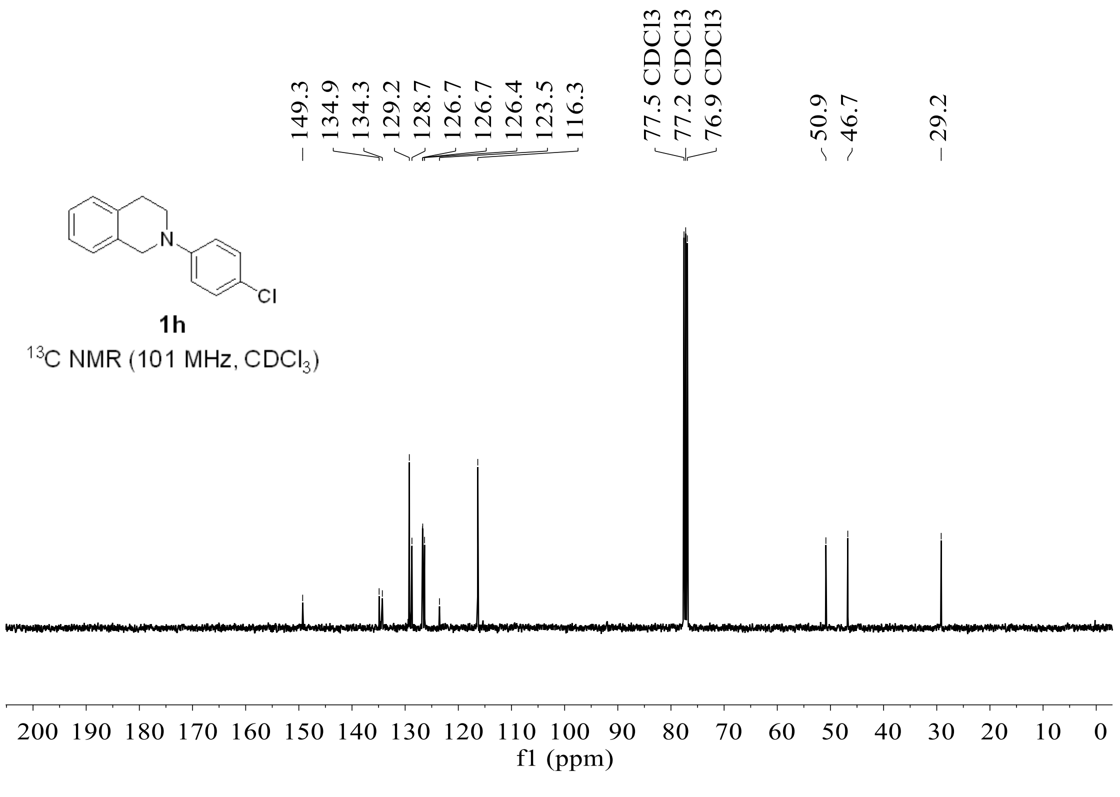

Figure S28. ^{1}H NMR and ^{13}C NMR spectra of compound **1h**.

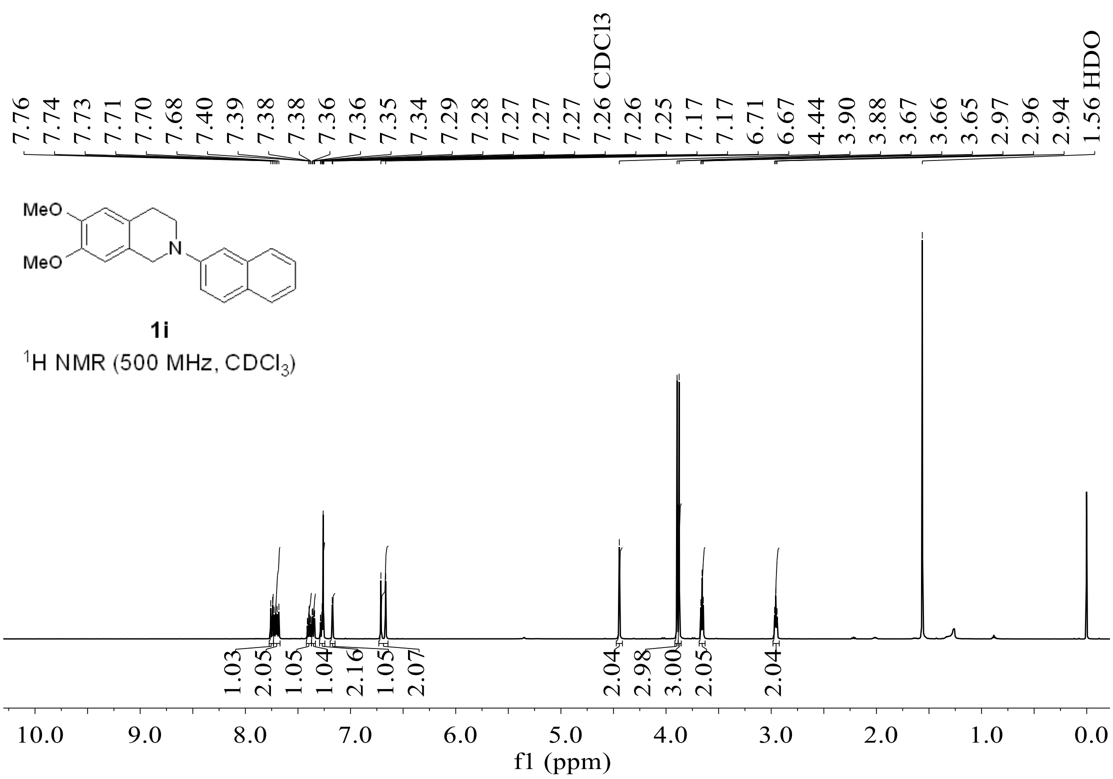

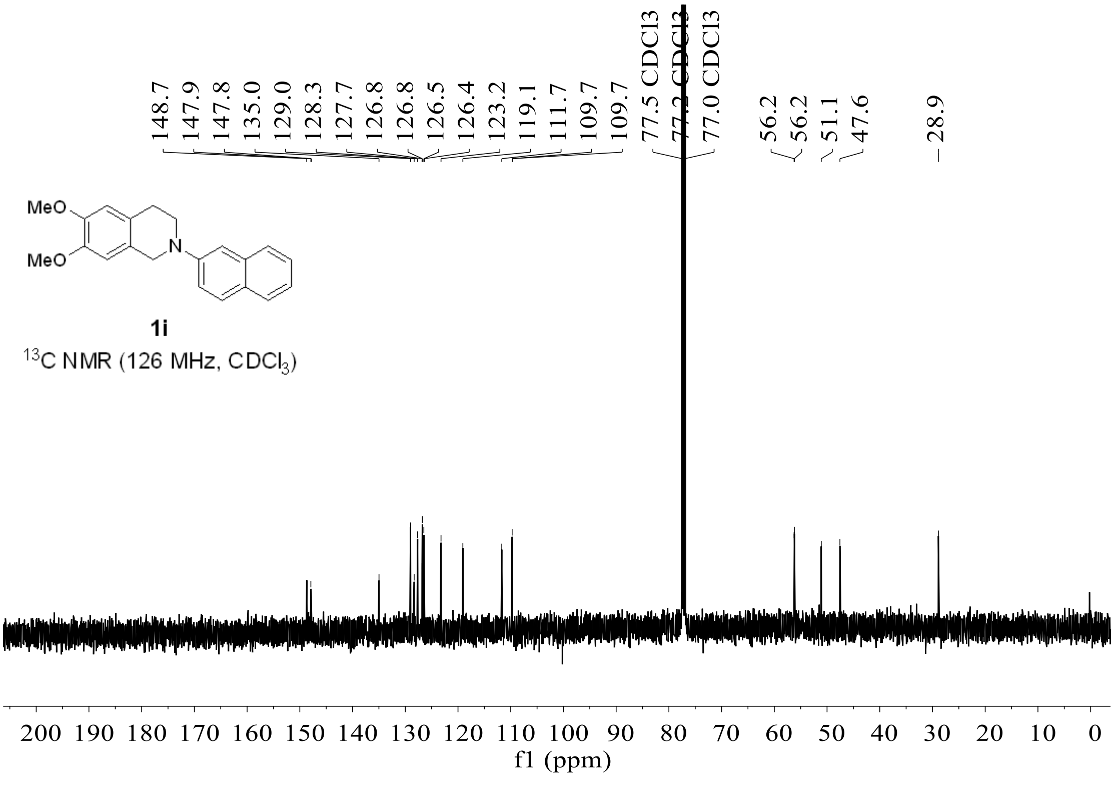

Figure S29. ^{1}H NMR and ^{13}C NMR spectra of compound **1i**.
